# Novel Cyclic Lipopeptide Antibiotics: Effects of Acyl Chain Length and Position

**DOI:** 10.3390/ijms21165829

**Published:** 2020-08-13

**Authors:** Signe Kaustrup Jensen, Thomas T. Thomsen, Alberto Oddo, Henrik Franzyk, Anders Løbner-Olesen, Paul R. Hansen

**Affiliations:** 1 Department of Drug Design and Pharmacology, Faculty of Health and Medical Sciences, University of Copenhagen, Universitetsparken 2, 2100 Copenhagen, Denmark; signekaustrupjensen@outlook.com (S.K.J.); albi.oddo@gmail.com (A.O.); henrik.franzyk@sund.ku.dk (H.F.); 2 Department of Clinical Microbiology, Rigshospitalet, Henrik HarpestrengsVej 4A, 2100 Copenhagen, Denmark; thomas.thomsen@bio.ku.dk; 3 Department of Biology, Section for Functional Genomics, University of Copenhagen, Ole MaaløesVej 5, 2200 Copenhagen, Denmark; lobner@bio.ku.dk

**Keywords:** cyclic lipopeptides, colistin, antimicrobial peptides, fatty acid, hydrophobicity

## Abstract

Multidrug-resistant bacteria are a global health problem. One of the last-resort antibiotics against Gram-negative bacteria is the cyclic lipopeptide colistin, displaying a flexible linker with a fatty acid moiety. The aim of the present project was to investigate the effect on antimicrobial activity of introducing fatty acid moieties of different lengths and in different positions in a cyclic peptide, S3(B), containing a flexible linker. The lipidated analogues of S3(B) were synthesized by 9-fluorenylmethoxycarbonyl (Fmoc) solid-phase peptide synthesis. Following assembly of the linear peptide by Fmoc solid-phase peptide synthesis, on-resin head-to-tail cyclization and fatty acid acylation were performed. The antimicrobial activity was determined against the ESKAPE pathogens, *Staphylococcus aureus*, *Klebsiella pneumoniae*, *Acinetobacter baumannii*, *Pseudomonas aeruginosa*, and *Escherichia coli*. Furthermore, hemolytic activity was determined against human erythrocytes. A total of 18 cyclic lipopeptides were synthesized and characterized. It was found that introduction of fatty acids in positions next to the flexible linker was more strongly linked to antimicrobial activity. The fatty acid length altered the overall hydrophobicity, which was the driving force for both high antimicrobial and hemolytic activity. Peptides became highly hemolytic when carbon-chain length exceeded 10 (i.e., C_10_), overlapping with the optimum for antimicrobial activity (i.e., C_8_–C_12_). The most promising candidate (C_8_)^5^ showed antimicrobial activity corresponding to that of S3(B), but with an improved hemolytic profile. Finally, (C_8_)^5^ was further investigated in a time-kill experiment.

## 1. Introduction

The inappropriate use of antibiotics has resulted in a global health crisis wherein multidrug-resistant bacteria often are encountered in healthcare-associated infections [1]. Especially worrying are the ESKAPE pathogens *Enterococcus faecium*, *Staphylococcus aureus*, *Klebsiella pneumoniae*, *Acinetobacter baumannii*, *Pseudomonas aeruginosa*, and *Enterobacter* spp. [2]. Currently, the treatment options are very limited, and the annual death toll caused by antimicrobial resistance is predicted to rise to 10 million by 2050 [3]. Therefore, the development of novel antimicrobial agents is of great importance. A recent survey of the preclinical antibacterial pipeline, ending May 2019, identified 407 preclinical trials, including direct-acting agents, antibodies and vaccines, phages and phage-related products, microbiota-modulating therapies, antivirulence approaches, antimicrobial potentiators, repurposed drugs, and immunomodulatory molecules [4]. Furthermore, siderophore-antibiotic conjugates such as cefiderocol constitute an interesting new class of antibacterial agents [5].

Antimicrobial peptides (AMPs) were discovered in the 1980s, and they have since received attention as potential antibiotic candidates [6]. AMPs are a part of the innate immune system of multicellular organisms, and show high cell selectivity and broad-spectrum activity against several bacterial species [7,8]. Furthermore, they also display numerous other properties, including wound-healing, anti-cancer activity, and cell recruitment [9]. Typically, AMPs are less than 40 residues in length, cationic, and contain approximately 50% hydrophobic residues. AMPs mainly act by disrupting the bacterial membrane [10], although intracellular targets have been reported [11]. In addition, Velkov et al. looked at the secondary mode of action cationic peptides against respiratory enzymes such as NDH-2 in the bacterial membrane [12].

However, the use of AMPs as therapeutics has been hampered by their susceptibility to proteases and low bioavailability [13]. Introduction of nonstandard amino acids into the AMP sequence may circumvent these shortcomings. The resulting peptidomimetics often retain the antimicrobial activity of the parent peptides and are stable to proteolytic breakdown [14]. An alternative strategy for increasing their stability is to cyclize the AMPs [15].

The cyclic lipopeptide antibiotics colistin (polymyxin E) [16] and daptomycin [17] are considered to be two of the last-resort drugs available for the treatment of multidrug-resistant Gram-negative and Gram-positive bacteria, respectively. These lipopeptides consist of a cyclic peptide displaying a flexible exocyclic linker with a fatty acid tail.

The cyclic antimicrobial peptide S3(B), containing a flexible linker but no lipid tail, was recently reported by our group [18]. This peptide is structurally similar to the nonapeptides developed by Vaara et al. from polymyxins [19]. However, S3(B) had activity against *S. aureus* and *P. aeruginosa*, but no activity against other Gram-negative species tested. It is known that the fatty acid chain, e.g., in colistin, is essential for conferring high antimicrobial activity [20]. Thus, the aim of the present work was to investigate the effect of introducing fatty acid moieties of different lengths and at different positions in the structure of S3(B), thereby designing lipopeptide analogues of S3(B).

**Figure 1:**
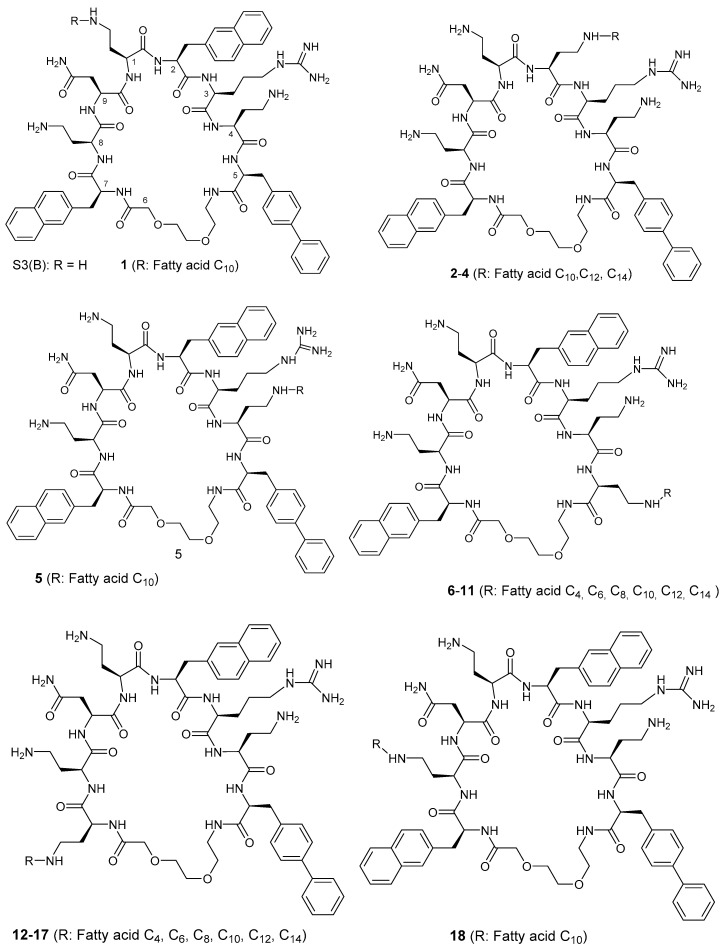
Structure of fatty acid S3(B) analogues studied in this paper.

**Scheme 1:**
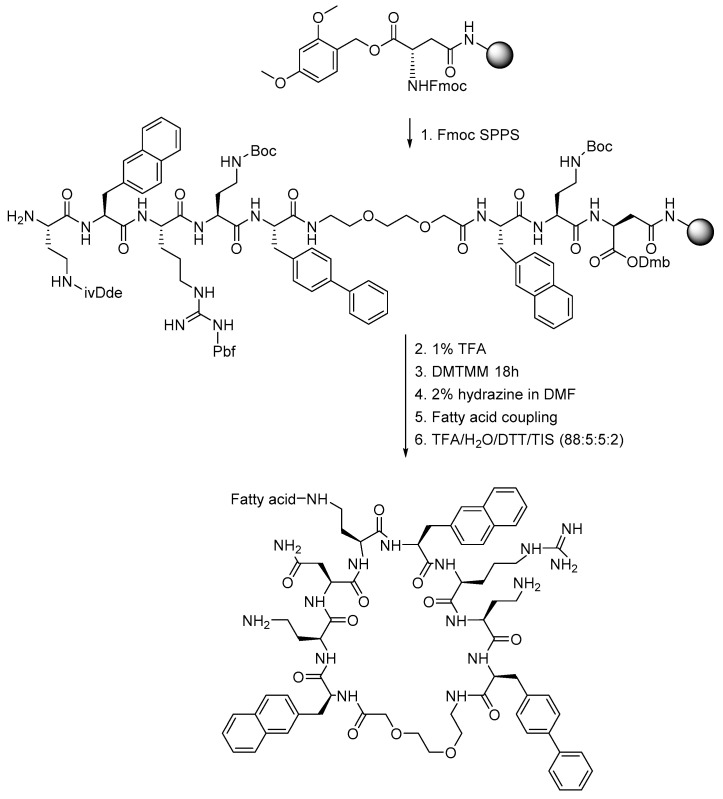
Strategy for synthesis of fatty acid S3(B) analogues (synthesis of cyclic lipopeptide 1 is shown).

These lipidated analogues of S3(B) were synthesized by Fmoc-based solid-phase peptide synthesis (SPPS), as depicted in Scheme 1. After reaching the desired sequence, on-resin head-to-tail cyclization and fatty acid acylation were performed. Peptides were purified by preparative HPLC, and further characterized by analytical RP-HPLC and MALDI-TOF MS. A total of 18 cyclic lipopeptides (Figure 1) were synthesized and investigated. It was found that attachment of fatty acids in positions next to the flexible linker was particularly strongly linked to high antimicrobial activity.

The antimicrobial activity was determined against the ESKAPE pathogens, while hemolytic activity was determined against human erythrocytes. The most promising candidate, (C_8_)^5^, based on its low hemolytic activity, was further investigated in a time-kill experiment.

## 2. Results and Discussion

### 2.1. Optimization of Synthesis

A total of 18 analogues of S3(B), acylated with fatty acids, were synthesized by Fmoc-based SPPS See Figure S1 and Tables S1–S3. The length and positioning (i.e., at Residues 1, 2, 4, 5, 7, and 8) of the fatty acid were varied to investigate how these parameters affect both the antimicrobial activity and hemolytic activity of the peptides. The fatty acid was either introduced directly onto an existing cationic residue displaying a temporarily protected amine functionality (i.e., Dab^1^, Dab^4^, or Dab^8^) or by replacing an already hydrophobic residue (i.e., Nal^2^, Bip^5^, or Nal^7^) with a Dab residue carrying the fatty acid moiety (also introduced via a two-step deprotection-acylation protocol). The peptides were named according to the position and length of the fatty acid, e.g., (C_10_)^1^ indicates that Dab^1^ is carrying a C_10_ fatty acid in the cyclic structure (Table 1).

Initially, our intention was to synthesize all six main peptide analogues displaying a C_10_ fatty acid (i.e., 1, 2, 5, 9, 15, and 18) as well as those displaying three longer fatty acids: C_12_, C_14_, and C_16_. However, after synthesis of the C_10_ analogues it was realized that solubility of the crude products was a problematic aspect. In particular, the highly hydrophobic peptides (C_10_)^1^, (C_10_)^4^, and (C_10_)^8^, in which a cationic side chain was replaced with a hydrophobic one, were found to possess low solubility. Consequently, these peptides were only synthesized with a C_10_ fatty acid attached. However, for the subseries of peptides, in which an aromatic residue was substituted by a Dab residue carrying a fatty acid, the analogues with elongated fatty acids, i.e., (C_12_)^2^/(C_14_)^2^, (C_12_)^5^/(C_14_)^5^, and (C_12_)^7^/(C_14_)^7^, were synthesized. These peptides also proved difficult to dissolve.

After peptide cyclization, we often observed a side product with a molecular weight equal to the cyclic peptide plus 139 Da. Judging from the HPLC retention times, this corresponded to a compound more hydrophobic than the respective target peptides. Possible explanations could be: (i) addition of a residual product from the ivDde group, as the ring in this structure corresponded to 139 Da; (ii) addition of Dmb to the peptide during TFA cleavage, or (iii) a side reaction with the coupling reagent DMTMM. We excluded that the side product was related to ivDde, since the side product was also observed in the less hydrophobic and unacylated peptide S3(B), in which the ivDde group had not been present during synthesis. This was confirmed by the observation that the peak only was present after cyclization, and not in the linear peptides.

In the literature, it is described that removal of the Dmb group via mild acetic conditions can lead to peptides with unacceptably low purity. To optimize, addition of scavengers to the cleavage cocktail was shown to improve trapping of reactive carbocations that give rise to undesired side reactions [21]. Therefore, it was reasonable to assume that the common impurity was related to this step in the synthesis. However, a side product involving addition of Dmb was ruled out since the molecular weight of the Dmb group is 151 Dalton and cyclization together with Dmb requires 133 Dalton. Instead, the side product was most likely related to the 4,6-dimethoxy-1,3,5-triazine part of the coupling reagent DMTMM, which corresponds to a mass of 139 Da. To alleviate this problem we used another cleavage cocktail that consisted of TFA/H_2_O/DTT/TIS (88:5:5:2) [22]. Dithiothreitol (DTT) is a nucleophilic scavenger originally used for reduction of disulfide bonds [23]. When including DTT in the cleavage cocktail, there was an improvement for all synthesized C_4_–C_8_ peptides in terms of solubility and purity. The crude peptides were now easily soluble even in water prior to purification. For the present peptides, introduction of fatty acids in different positions changed two important parameters: the positive net charge of the molecule and its hydrophobicity. Overall, peptides (C_10_)^1^, (C_10_)^4^, and (C_10_)^8^ were highly hydrophobic because C_10_ was attached directly onto an already existing Dab residue. Therefore, these peptides were composed of three hydrophobic aromatic residues and one C_10_ chain, and had a positive net charge of +3 at physiological conditions. In contrast, the peptides (C_n_)^2^, (C_n_)^5^, and (C_n_)^8^ had the fatty acid attached to a Dab residue instead of Nal or Bip. Thus, these subseries of peptides contained only two hydrophobic aromatic residues besides the fatty acid moiety, thereby being less hydrophobic than the other main subseries where the fatty acids were introduced as an additional hydrophobic moiety. Additionally, for these peptides, the positive net charge was +4 similar to that of S3(B), displaying three hydrophobic aromatic residues and no fatty acid. Peptide names, sequences, hydrophobicity, and data obtained from the antimicrobial and hemolytic experiments are listed in Table 1. Since none of the analogues showed activity against *K. pneumoniae* (MIC 64 μg/mL), this bacterium is not discussed further.

#### 2.1.1. Composition of the Growth Media in MIC Determinations

In the original report on S3(B), MIC experiments were carried out by using non-cation-adjusted MHB-I media. To this, bovine serum albumin (BSA) and acetic acid were added. The rationale for choosing a medium without cations is that cation-adjusted media (MHB-II) in some cases causes substantial inhibition of the activity of cationic peptides. Furthermore, some peptides are at risk of precipitation when diluted into MHB medium, which can be avoided via the addition of BSA and acetic acid [24]. However, the use of MHB-II is favorable to imitate the in vivo conditions as much as possible. Both media were tested with and without the addition of BSA and acetic acid by trial and error. The use of pure MHB-II was compatible with S3(B) and its fatty acid analogues, and therefore was chosen for the MIC determinations. Noticeably, MHB-I with BSA and acetic acid led to precipitation of lipopeptides in the present work.

#### 2.1.2. Fatty Acid Positioning Affects the Antimicrobial Activity

To investigate how the positioning of the fatty acid influence antimicrobial activity, peptides with similar structures (number of hydrophobic residues and charge) were compared. The cyclic lipopeptides were screened against wild-type isolates belonging to the ESKAPE group of pathogens (see Section 3). We included *E. coli* in the screen as a representative of Enterobacteriaceae. Overall, there were no differences in antimicrobial activity when comparing the MICs determined for (C_10_)^1^, (C_10_)^4^, and (C_10_)^8^. In contrast, there were some indications that the positioning of the fatty acid had importance when hydrophobic aromatic residues were substituted with a modified Dab residue. As shown in Table 1, the main difference between insertion of fatty acid tails in Positions 2 and 7 was the surrounding residues. In Position 2, the fatty acid was adjoined by two cationic residues, whereas the flexible linker was next to the fatty acid in Position 7. Otherwise, the overall sequences of (C_n_)^2^ and (C_n_)^7^ were identical. For the MICs, significant differences were observed against all bacteria. Compounds (C_12_)^2^ and (C_12_)^7^ were especially interesting: the antimicrobial activity was reduced to one-fourth when C_12_ was inserted in Position 2 as compared to Position 7. Because the fatty acid position was the only variable, it appears that attachment to some positions in the macrocycle gave rise to higher antimicrobial activity. This observation was further confirmed by the MICs determined for (C_n_)^5^. When a fatty acid moiety was inserted in Position 5, it was adjoined by a single cationic residue and the flexible linker, as when placed in Position 7. Interestingly, the MICs for all (C_n_)^5^ peptides (*n* = 4, 6, 8, 10, 12, and 14) were similar to those found for the corresponding (C_n_)^7^ peptides (*n* = 4, 6, 8, 10, 12, and 14), even though the peptides had slightly different sequences. It could therefore be hypothesized that it is favorable to introduce a fatty acid in the vicinity of a hydrophilic, flexible linker.

In a study concerning lipidated cyclic γ-AApeptides, Li et al. found that attachment of fatty acid tails directly to the rigid macrocyclic structure only gave rise to analogues that possessed weak antimicrobial activity [25]. The reason for this could be that the lipid tail had limited flexibility when attached to the ring structure, thus reducing its ability to insert efficiently into the bacterial membrane. When the lipid tail was moved away from the cyclic structure by insertion of at least two residues between the macrocycle and the fatty acid, the antimicrobial activity was enhanced substantially. This indicated that incorporation of flexible parts into macrocycles might possibly confer less constriction to the movement of the lipid tail [25]. A flexible linker such as 8-amino-3,6-dioxaoctanoic acid (O_2_Oc) also creates a discrete region of flexibility in an otherwise constrained macrocycle. Therefore, it was reasonable to assume that the flexible linker modified the conformation of the region around it, and thus facilitated the lipid tail to anchor deeply into the bacterial membrane. Furthermore, consistent with Li et al., addition of fatty acids in more rigid positions in the S3(B) analogues, e.g., in Position 2, did not lead to enhanced antimicrobial activity. Therefore, it is suggested that a certain degree of flexibility around the fatty acid moiety is essential to confer antimicrobial properties.

#### 2.1.3. The Influence of Cationic Residues in AMPs

When C_10_ fatty acids were attached at Positions 1, 4, and 8 in the macrocycle, the net positive charge was reduced as compared to that of the lead structure. For all three analogues, the activity against *E. coli* and *P. aeruginosa* was completely lost (MIC > 64 μg/mL). Based on this observation, it is hypothesized that reducing the number of cationic residues in the AMPs led to a decreased electrostatic interaction between the peptide and the negatively charged bacterial lipopolysaccharide (LPS). Several studies have investigated the influence of charge on antimicrobial activity of AMPs. Giangaspero et al. showed that decreasing or increasing the net positive charge of AMPs either reduced or enhanced the antimicrobial activity, respectively [26]. Likewise, Dathe et al. showed that increasing the net positive charge from +3 to +4 or from +4 to +5 (threshold) gradually increased the antimicrobial activity [27]. It is therefore reasonable that the decrease in charge from +4 to +3 in some of our fatty acid analogues could be an explanation for the loss of activity. However, the activity against *S. aureus* and *A. baumannii* (8–16 μg/mL) determined for (C_10_)^1^, (C_10_)^4^, and (C_10_)^8^ was comparable to that of S3(B). Thus, *E. coli* and *P. aeruginosa* are clearly more sensitive to alterations of the net charge of certain AMPs, possibly because this leads to weaker electrostatic interactions with bacterial membranes.

#### 2.1.4. The Influence of Fatty Acid Length

To investigate the effect of attaching fatty acids to a macrocycle, fatty acid tails of different lengths were used. When the carbon chain was elongated, the hydrophobicity of the peptide was increased gradually. Herein, the hydrophobicity is expressed as the percentage of mobile phase B at the peak elution time on an RP-HPLC system. As expected, all lipidated analogues displayed relatively high hydrophobicity due to the presence of a fatty acid tail.

**Figure 2:**
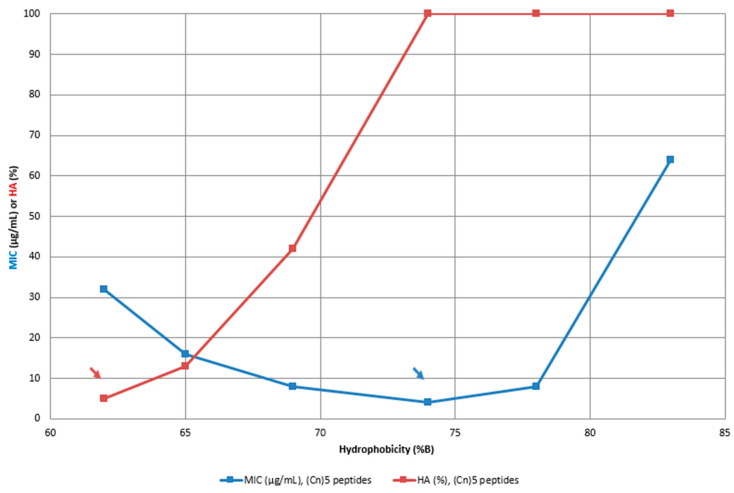
Relationship between hydrophobicity and antimicrobial activity (MIC, μg/mL) and hemolytic activity (% hemolysis at 150 μM) of the (C_n_)^5^ series of peptides. Data shown are obtained against *P. aeruginosa* and human erythrocytes. Arrows indicate the hydropmichobicity optima for MIC (minimum inhibitory concentration) and HA, the two optima being displaced.

Generally, opposing trends for antimicrobial and hemolytic activity were found when the fatty acid length was increased. An example is shown in Figure 2.

The antimicrobial activity against *P. aeruginosa* (blue curve) and hemolytic activity at 150 μM (red curve) for the (C_n_)^5^ peptides are illustrated as a function of increasing hydrophobicity (via elongating the fatty acid from C_4_ to C_14_). It is generally accepted that increasing hydrophobicity of AMPs typically leads to enhanced hemolytic properties. In the same way, some hydrophobicity of an AMP is required for bacterial membrane permeabilization, and it usually confers improved antimicrobial activity [28].

### 2.2. Antimicrobial Activity

As illustrated in Figure 2, it was clear that an optimum in fatty acid chain length existed. When the lipid tail was shorter than this most effective length (i.e., C_10_ with an optimal MIC of 4 μg/mL against *P. aeruginosa*; 4–16 μg/mL against other bacteria) the potency was lowered, gradually reaching MICs of 32 μg/mL or higher. In contrast, when the lipid tail was elongated beyond C_10_, the activity gradually decreased to a MIC of 64 μg/mL. When comparing the MICs for all tested species, the antimicrobial activity of the fatty acid modified analogues was lowered significantly (to MICs of 16 μg/mL or higher) when fewer than eight or more than twelve carbon atoms were present in the lipid tail. These observations may possibly be interpreted as a too-short fatty acid tail being unable to penetrate the bacterial membrane, consequently causing lack of activity. In contrast, tails longer than the maximum effective length could induce aggregation (or adsorption to test plates) and subsequently inactivation of AMPs due to strong hydrophobic interactions between the fatty acid tails [29]. Thus, the best activity was obtained when C_10_ fatty acids were attached at Positions 5 or 7, i.e., to give lipopeptides (C_10_)^5^ and (C_10_)^7^.

Importantly, the present results were in agreement with the relationship between peptide hydrophobicity and antimicrobial activity observed in a study conducted by Chen et al. [28]. In that study, alteration in hydrophobicity was achieved via amino acid substitutions, while in the present work hydrophobicity was varied by modification with fatty acids. Therefore, it is difficult to assess whether the antimicrobial activity mainly is determined by the change in overall hydrophobicity or whether the ability of the fatty acid moiety to anchor the molecule in the bacterial membrane also contributed. No differences were seen when the antimicrobial activity of S3(B) is compared to the activity of the equally hydrophobic analogue (C_8_)^7^. This indicates that the length of fatty acid contributed to the peptide hydrophobicity and amphipathic structure, whereas further ability to enhance activity through membrane anchoring appeared to be of minor importance for this class of cyclic lipopeptides. In other studies, it has been shown that membrane binding only is slightly increased for lipopeptides containing a single fatty acid moiety. Thus, the lipid tail’s contribution to the overall binding enthalpy may be negligible. In contrast, when two fatty acid chains were attached to a peptide, the lipid tails were firmly anchored in the membrane [30].

#### 2.2.1. Hemolytic Activity

Hemolysis of red blood cells is often used as a preliminary assessment of antimicrobial peptides [31]. The lowest hemolytic activity was found for (C_4_)^5^, and it was gradually increased with increasing fatty acid chain length. Increasing the fatty acid length from C_8_ to C_10_ led to a pronounced increase in the hemolytic properties (Table 1). The critical length for fatty acid to confer hemolytic properties to the lipopeptide was found to be more than eight carbon atoms. This tendency was consistent with data obtained for the (C_n_)^7^ peptides as well.

**Figure 3:**
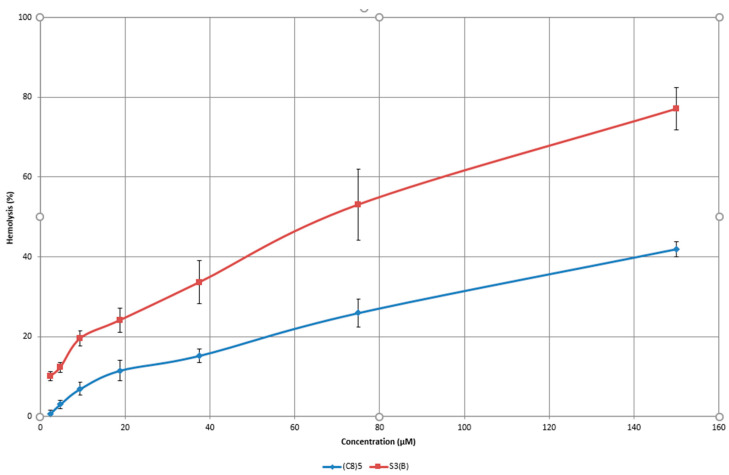
Hemolysis as a function of peptide concentration. In the present study, the peptide (C_8_)^5^ (blue curve) showed significant lower toxicity toward erythrocytes than S3(B) (red curve) in all tested concentrations (2.35–150 μM). The results are presented as mean ± standard deviations (*n* = 3).

With regards to hydrophobicity, only the C_4_-, C_6_-, and C_8_-modified peptides were less hydrophobic than the original S3(B), whereas the hydrophobicity was increased when the fatty acid length was extended further. In addition, only the C_4_-, C_6_-, and C_8_-modified peptides had lower hemolytic activity than S3(B). The peptides that were more hydrophobic than S3(B), i.e., all lipopeptides with fatty acids longer than eight carbon atoms, were found to be highly hemolytic, with induction of 100% hemolysis at 150 μM. When the hydrophobicity was too pronounced, the AMPs were not able to differentiate between cells based on hydrophobic interactions. Because S3(B) naturally was relative hydrophobic and hemolytic with an HD_50_ of 69 μM (Figure 3), more hydrophobic analogues may have been able to penetrate deeper into the hydrophobic core of human erythrocytes, thereby causing lysis of cells [28].

Because of the different optima for antimicrobial and hemolytic activity based on peptide hydrophobicity, choosing a peptide candidate for further studies was a compromise. Here, nontoxic peptides were without antimicrobial activity, while the peptides showing the best antimicrobial profile were too hemolytic. Therefore, the peptide with the best antimicrobial activity combined with an acceptably low human toxicity was considered to meet the requirements for further analysis. As the antimicrobial activity of all (C_n_)^5^ and (C_n_)^7^ peptides generally was very similar, the hemolytic activity was the determining factor. When inspecting both antimicrobial and hemolytic activities, the C_8_-modified peptides were considered the most promising analogues. Since the hemolytic activity of (C_8_)^5^ was lower than that of (C_8_)^7^, the former was selected for further investigation.

#### 2.2.2. (C_8_)^5^: The Most Promising Lipopeptide Analogue of S3(B)

Overall, the results indicated that the most promising S3(B) lipopeptide analogue was (C_8_)^5^. This analogue showed antimicrobial activity corresponding to that of S3(B) against all tested bacteria, with highest potency observed against *S. aureus* and *P. aeruginosa* with MICs of 8 μg/mL. However, a significantly lower hemolytic activity was observed for (C_8_)^5^ (HD_50_ > 150 μM) as compared to 69 μM for S3(B). As discussed above, the optimum in antimicrobial activity did not co-occur with low hemolytic activity, making (C_8_)^5^ the best choice out of the synthesized peptides. Furthermore, we expected (C_8_)^5^ to be relatively stable in serum, since cyclic peptides are significantly more resistant to proteolytic breakdown than linear peptides.

#### 2.2.3. Hemolytic Activity: (C_8_)^5^ Versus S3(B)

Differences in hemolytic activity for (C_8_)^5^ and S3(B) were observed across the entire investigated concentration range 2.35–150 μM. This is illustrated in Figure 3. As expected, the degree of hemolysis was dependent on the peptide concentration; more erythrocytes were affected and lysed when increasing amounts of peptide molecules were available. Therefore, the curves in Figure 3 represent the typical pattern for a hemolytic profile [31]. An explanation for the less hemolytic profile for (C_8_)^5^ as compared to that of S3(B) might be the peptide hydrophobicity, even though the difference was small. When the hydrophobicity was slightly increased, highly hemolytic peptides were obtained, suggesting that a threshold in hydrophobicity close to that of (C_8_)^5^ existed.

#### 2.2.4. Time-Kill Experiment of (C_8_)^5^ Against *P. aeruginosa*

The MIC constitutes a limited measure for antimicrobial activity. It indicates whether or not AMPs in different concentrations are able to prevent visible growth of bacteria. However, it does not provide information about the time course of the antimicrobial effect or about how effective the agent is, i.e., the fraction of bacteria being killed during the period of treatment. Therefore, a time-kill experiment of (C_8_)_5_ was performed against *P. aeruginosa*. Using different peptide concentrations of 1 × MIC (8 μg/mL) and 5 × MIC (40 μg/mL), important information about the rate and extent of bacterial killing as a function of concentration was generated. For comparison, colistin 5 × MIC (2.5 μg/mL) and S3(B) 1 × MIC (8 μg/mL) were used as reference compounds.

**Figure 4:**
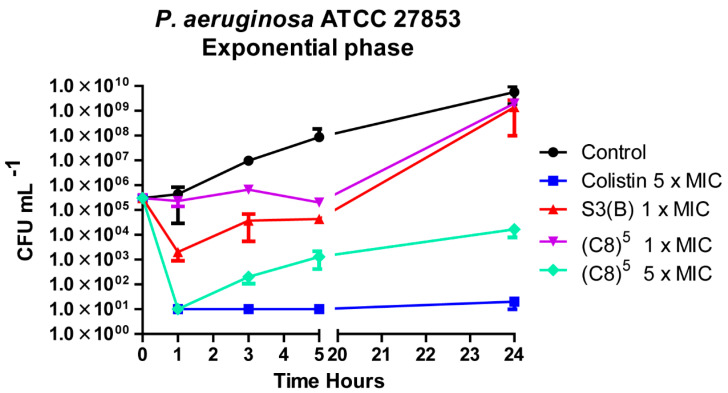
Time-kill curves for (C_8_)^5^ against *P. aeruginosa* at different concentrations; 1 × MIC and 5 × MIC. Data obtained for S3(B) and colistin are included as controls. The results are presented as mean ± standard deviations (*n* = 3).

As illustrated in Figure 4, the data obtained for (C_8_)^5^ clearly showed that the peptide exhibited concentration-dependent killing against *P. aeruginosa*. Thus, when treated for 1 h, the cell count was reduced differently; a 4.5-log reduction was observed at 5 × MIC compared to 1 × MIC. This corresponded to killing of >99.9% of the bacteria in the original inoculum. In contrast, when treating with a concentration equal to the MIC, the bacterial cell count did not change over the first 5 h, indicating a more bacteriostatic effect at this concentration. For comparison, 2.5-log and 4.5-log reductions were found within the first hour of treatment for S3(B) and colistin, respectively. The data obtained for S3(B) and colistin were comparable to previous data for the two compounds [18]. However, the difference in effect between S3(B) and (C_8_)^5^ indicated that the lipopeptide derivative was less bactericidal than the original cyclic peptide. The high-dose treatment with (C_8_)^5^ showed high initial bactericidal activity, followed by a plateau, and finally, slow regrowth seemed to occur. This effect was unexpected. There was a clear concentration-dependent activity of the peptide, which was expected due to the fact that AMPs mainly act through binding to the bacterial membrane, and not through a specific target, e.g., receptors which can be saturated because of occupation of binding sites. When the peptide concentration is increased, more molecules are available to bind to the bacterial membrane. Therefore, more cells can potentially be affected and killed as compared to treatment with a lower peptide concentration.

It seemed likely that a concentration equal to the MIC of (C_8_)^5^ was able to maintain the bacterial concentration of the original inoculum for 5 h. However, this effect was transient, and after 24 h, the cell count was increased to the same level as the growth control (~10^10^ CFU/mL). This transient effect was also seen for S3(B), but with an initial bactericidal phase. After five hours of treatment with 5 × MIC, the bacterial concentration was similar to the concentration obtained upon exposure to 1 × MIC of (C_8_)^5^. Because the antimicrobial effect is highly dependent on the number of viable cells relative to the amount of available drug molecules, variation is commonly observed.

Additionally, the MIC determinations were performed using different growth conditions than during the time-kill experiment. In the latter, the bacterial suspension was shaken and aerated during growth, thereby ensuring optimal growth conditions for the bacteria. Therefore, it is possible that the antimicrobial concentration determined in the MIC assay differed from the concentration needed to kill bacteria when more favorable growth conditions are provided. Colistin was the only peptide that retained a high bactericidal activity over 24 h. No growth was observed in any of the colistin-treated samples. When no growth of bacteria was observed, the CFU/mL was therefore set at the detection limit of 10 CFU/mL. The remaining peptide preparations were not able to prevent growth completely for more than one hour, as regrowth of *P. aeruginosa* then occurred. Applying 5 × MIC of (C_8_)^5^ gave a slightly increased duration; however, cells regrew to 10^4^ after 24 h when the experiment was terminated.

Overall, the antimicrobial activity of (C_8_)^5^ was concentration-dependent, with high killing efficacy when an appropriate concentration was used. However, it was only possibly to maintain the high bactericidal effect (>99.9% killing) for one hour.

## 3. Materials and Methods

### 3.1. Chemistry

TentaGel^®^ S RAM resin, TFA, piperidine, Fmoc-8-amino-3,6-dioxaoctanoic acid, and all Fmoc-protected l-amino acids were purchased from Iris-Biotech GmbH, Marktredwitz, Germany DIEA, triisopropylsilane, PBS, melitin, 4-(4,6-dimethoxy-1,3,5-triazin-2-yl)-4-methylmorpholiniumtetrafluoroborate (DMTMM.BF_4_), and 2% hydrazine in DMF and fatty acids were from Sigma-Aldrich (St. Louis, MO, USA); HOAt (1-hydroxy-7-azabenzotriazole) and HATU (1-[*bis*(dimethylamino)methylene]-1*H*-1,2,3-triazolo [4,5-b]pyridinium-3-oxid hexafluorophosphate, *N*-[(dimethylamino)-1*H*-1,2,3-triazolo-[4,5-b]pyridin-1-ylmethylene]-N-methylmethanaminium hexafluorophosphate *N*-oxide), were from GL Biochem (Shanghai, China) DMF (dimethylformamide, synthesis grade), DCM (dichloromethane, optical grade), and ACN (acetonitrile, optical grade) were from VWR (Copenhagen, Denmark). Disposable 5 mL polypropylene reactors fitted with PTFE filters were acquired from Thermo Scientific (Copenhagen, Denmark).

### 3.2. Peptides

Peptides were purified via preparative RP-HPLC on a preparative WatersTMXBridgeTM BEH130 C_18_, HPLC column (5 μm; 10 × 250 mm) equipped with a WatersTM Cartridge Holder PKG (10 × 10 mm). MicroflexTM (Bruker Corporation, Bremen, Germany). FlexControl software (Bruker Daltonik GmbH, Bremen, Germany) was used to obtain the spectra, and the data were processed using flexAnalysis (Bruker Daltonik GmbH). All reagents and solvents were used without further purification.

### 3.3. Microbiology and Hemolysis

Cation-adjusted Mueller-Hinton broth (MHB-II) media (BD BBLTM Beef extract powder, BD BactoTMCasamino acids, and DIFCO soluble starch was from Becton, Dickinson and Company©, Franklin Lakes, NJ, USA). MHB media supplemented with 0.2% BSA was from Sigma Aldrich (St. Louis, MO, USA) and 0.01% acetic acid from VWR^®^. The 96 well plate cell-culture cluster, round-bottom, polypropylene plates, and V-shaped 96 well polypropylene plates were purchased from Corning^®^ Inc., Costar^®^, Corning, NY, USA. Microseal^®^ film was obtained from Bio-Rad Laboratories, Inc., Hercules, CA, USA. A VersaMaxTM Tunable Microplate Reader (Molecular Devices LLC, Sunnyvale, CA, USA) was used and the data were evaluated on a Softmax^®^ Pro (Molecular Devices LLC). [32]

### 3.4. Peptide Synthesis

Peptides were synthesized according to standard Fmoc-based SPPS procedures, which were performed on a TentaGel S RAM resin (loading 0.24 mmol/g). Overall, the synthesis consisted of cycles of Fmoc deprotection, washing, amino acid coupling, and washing. Afterwards, macrocyclization, fatty acid acylation, and finally cleavage from the resin were performed [18].

### 3.5. Fmoc Deprotection

The Fmoc-protected RAM linker was deprotected using 20% piperidine in DMF. A volume of 3 mL was transferred into the syringe, which was allowed to stand for 4 min. Afterwards, the syringe was drained and washed with DMF (2×). The procedure was repeated twice followed by a full wash of the resin: DMF (4×), DCM (3×), and DMF (4×).

### 3.6. Initiation and Preparation of Syringes

The resin (300 mg) was weighed directly into a disposable 5 mL syringe fitted with a PTFE filter. The piston was inserted into the syringe and pushed just above the resin. DMF was drawn into the syringe and it was left to swell for at least 2 h. After swelling, the syringe was drained, the piston was removed, and the syringe was placed in a suction plate connected to a 2 L Büchner flask and a vacuum pump.

### 3.7. Coupling of Amino Acids

HOAt and HATU were weighed off separately and were dissolved in DMF as stock solutions (0.4 M). Four molar equivalents of amino acids and coupling reagents and eight equivalents of DIEA were used. The amino acids were weighed directly into appropriate tubes, and were dissolved in the HOAt stock solution. The amino acid solutions were kept in the freezer until use. Before coupling of an amino acid, HATU was transferred into the tube containing amino acid followed by addition of DIEA. The tube was gently shaken (color change to yellow) and was drawn up into the syringe. The syringe was wrapped in alufoil and placed on a shaker for 1.5 h at room temperature. Between double couplings, the resin was washed with DMF (2×). Between coupling of different amino acids, the resin was washed with DMF (6×), treated with 20% piperidine in DMF and washed with DMF (10×). The elongation with amino acids was continued until the final linear peptide was assembled.

### 3.8. Peptide Macrocyclization

To allow cyclization, the resin was treated with 1.5% TFA in DCM for 5 min to remove the Dmb group. This was repeated eight times in total, or more if necessary; through the treatments, the solution had to change color into red, indicating that the Dmb removal was driven to completion. The resin was washed with DMF (5×), DCM (5×), and EtOH (5×) following by lyophilization overnight. The lyophilized resin was allowed to swell in DMF for at least 2 h before initiation of the cyclization. DMTMM (3 equivalents) was weighed directly into an Eppendorf tube and was dissolved in a small amount of DMF. Six equivalents of DIEA was added to the solution, which then was drawn into the syringe. The peptide was left for cyclization overnight on a shaker. Afterwards, the resin was washed with DMF (5×). The procedure was repeated with 3 equivalents of DIEA. After completion of the cyclization, the resin was washed with DMF (3×), DCM (3×), and EtOH (5×), followed by lyophilization overnight.

### 3.9. Fatty Acid Acylation

To allow acylation, the ivDde group was removed. The lyophilized resin was allowed to swell in DMF for 30 min. Deprotection was accomplished via three treatments with a freshly prepared solution of 2% hydrazine in DMF. To each syringe, 3 mL (equal to 10 mL per g resin) of the solution was drawn up, and then the syringe was placed on a shaker for 15 min. The resin was washed with DMF (2×), and the procedure was repeated twice. After the third treatment, the resin was washed with DMF (3×) and DCM (3×), and it was allowed to dry completely. Fatty acids were weighed and dissolved in HOAt stock solution. The procedure for coupling of fatty acids was identical to the coupling of amino acids described above. Double couplings were performed, in which one of the couplings was carried out overnight. Finally, the resin was washed with DMF (3×), DCM (3×), and EtOH (5×) and lyophilized for at least 1.5 h.

### 3.10. Peptide Cleavage

The peptide was cleaved from the resin using a freshly prepared cleavage cocktail consisting of TFA/H_2_O/TIS (95:2.5:2.5 (*v*/*v*)) or TFA/H_2_O/DTT/TIS (88:5:5:2). Three milliliters of the cocktail was drawn into the syringe, the syringe was capped with a pressure cap, and the syringe was left on a shaker for two hours. Afterwards, the cleavage solution was pushed into a 5 mL cryotube. The piston was removed from the syringe and the resin was washed with cleavage cocktail, and then the eluate was collected in the cryotube. The collected cleavage solution was evaporated using a gentle stream of nitrogen until approximately 300 μL was left. An amount of 4 mL cold diethyl ether was added to the remaining solution, resulting in precipitation of the peptide. The tube was shaken gently and centrifuged (2000 rpm) for 6 min. The supernatant was removed and the procedure by adding diethyl ether was repeated twice using 2000 rpm and 4000 rpm, respectively. The cryotube was left to stand in the fume hood, allowing evaporation of the residual ether. After obtaining a dry crude, this was dissolved in a minimum freeze-drying solution (10% ACN and 0.1% TFA in water) and was left in the freezer (−80 °C) until it was completely frozen. The peptide (TFA salt) was lyophilized overnight to obtain a fluffy white solid.

### 3.11. Minimum Inhibitory Concentration Determination

The MIC experiments were performed according to a protocol adapted from Wiegand et al. [24], with minor modifications. The MICs were determined in triplicate. The peptides were dissolved in Milli-Q water to produce 10 mg/mL stock solutions in small glass containers. The bacterial isolates to be tested were streaked onto agar plates to obtain single colonies, and the plates were incubated at 37 °C for 18–24 h. The strains tested were *S. aureus* ATCC 29213, *P. aeruginosa* ATCC 27853, *E. coli* ATCC 25922, *A. baumannii* ATCC 19606, and *K. pneumonia* ATCC 13883. Test compounds were prepared in 2-fold dilutions in 50 μL volumes in polypropylene microtiter plates. Bacterial cultures grown to OD_600_ = 0.1–0.4 were diluted to a desired OD_600_ of 0.001–0.002 corresponding to 1 × 10^6^ CFU/mL. Next, 50 μL bacterial suspension was added to the microtiter plates, giving a final bacterial inoculum of 5 × 10^5^ CFU/mL per well. The plates were incubated at 37 °C for 18–24 h. To verify the CFU, 10 μL from the positive control was transferred to 990 μL 0.9% NaCl. From this, a 10-fold dilution series was made; 100 μL of the diluted bacteria was plated onto three LB plates, and these were incubated at 37 °C for 18–24 h. A cell count of 3–7 × 10^5^ CFU was considered acceptable. The MIC values were visually evaluated, and the MIC was set to the lowest concentration with visible growth based on triplicate measurements. Colistin and vancomycin were used as control antibiotics.

### 3.12. Hemolysis

The hemolysis experiments were performed according to the protocol described in Reference [31]. The peptides were tested at concentrations from 2.35–150 μM, using melittin as positive control and PBS as negative control. One premade tablet of PBS was dissolved in 200 mL Milli-Q water. The solution was kept in the refrigerator for a maximum of two days. The day before the test, the positive control wells of the 96 well polypropylene plates were precoated with 150 μL of melittin 5 μM solution in PBS. The solution was prepared in Protein LoBind Eppendorf tubes. The day of the test, the solution was discarded and the wells were washed three times with 150 μL PBS. A melittin 2.50 μM solution in PBS was prepared in Protein LoBind Eppendorf tubes—500 μL for every plate to be used. The mass of the peptide needed to reach a final concentration of 300 μM (to start testing down from 150 μM) was calculated, since a volume of 500 μL was desirable. Because the peptides were obtained as TFA salts during cleavage, this had to be taken into consideration in the calculations. The peptides were dissolved in PBS and tested in triplicate.

In a 96 well polypropylene plate, 150 μL of peptide solution was transferred to three wells in Row A. Therefore, each plate could accommodate up to four peptides. A 2-fold serial dilution of the peptides was obtained by transferring 75 μL of the solutions from Row A to the corresponding wells in Row B, mixing a couple of times. From Row B, 75 μL was transferred to Row C and so on until Row G, where 75 μL was discarded after mixing. Volumes of 75 μL melittin (2.50 μM) was transferred to the positive control wells in H1–H6. Preparation of the RBC suspension: 1 mL of whole blood was transferred to a cryotube with 3 mL of PBS, and then it was gently mixed and centrifuged for 8 min at 3000 rpm. The supernatant was discarded, and the process was repeated twice; with 4 mL at 3000 rpm and 4 mL at 4000 rpm, respectively. From the final RBC pellet, 40 μL was transferred to 8 mL of PBS (multiplied by the number of test plates needed). This corresponded to a 0.5% *v/v* RBC suspension. The suspension was gently mixed and transferred into a CAPPOrigami reservoir. Using a multichannel pipette, 75 μL of the suspension was transferred into each well and mixed in the direction of increasing concentration. The control wells were mixed separately. The plates were covered with BioradMicroseal foil and incubated at 37 °C for 1 h. The plates (maximum two) were centrifuged for 10 min at 4000 rpm, followed by transferring of supernatant (60 μL) from each well into the corresponding wells in a clear ELISA plate. The absorbance (A) of each well was measured at λ = 414 nm using the software program Softmax(R) Pro.

### 3.13. Time-Kill Kinetics

Time-kill experiments were performed on balanced exponentially growing cultures of *P. aeruginosa* ATCC 27853. We used balanced cultures as described in Oddo et al. [33]. Briefly, overnight cultures were prepared in MHB-II broth at 37 °C with shaking. Overnight cultures were back-diluted and grown exponentially for no less than 8–10 generations before experimentation. When experiments were done, exponentially growing cultures were back-diluted to an OD_600_ = 0.0005 approximately 5 × 10^5^ CFU, into fresh MHB-II heated to 37 °C. Samples (250 μL) were taken at time points 0, 1, 3, 5, and 24 h. Samples were spun down at 8000× *g* for 5 min and washed in 1 mL of NaCl 0.9% before being spun down again and re-suspended in 250 μL NaCl 0.9%. From this, 10-fold dilution series were prepared and 10 μL samples were spot-plated in triplicate onto LB-agar. From the undiluted sample, 100 μL samples were also plated, i.e., giving a detection limit of 10 CFU/mL.

## 4. Conclusions

A total of 18 fatty acid analogues of the flexible cyclic antimicrobial peptide S3(B) were synthesized in the present study. The peptides were cyclized head-to-tail, which generally makes peptides constricted and rigid molecules. However, by introducing a flexible residue (i.e., O_2_Oc) into the structure, it was possible to create a localized flexible region. Colistin and other cyclic lipopeptides naturally contain fatty acid tails that are essential for their antimicrobial activity. Therefore, the aim of this work was to investigate how the antimicrobial and hemolytic activity was affected by introduction of fatty acid chains of different lengths and in different positions in the macrocyclic structure of S3(B).

Two main types of analogues were synthesized: (i) highly hydrophobic peptides in which a fatty acid moiety was attached to an existing Dab residue: (C_n_)^1^,(C_n_)^4^, and (C_n_)^8^_,_ and (ii) peptides in which a Dab carrying a fatty acid chain was incorporated instead of an aromatic residue: (C_n_)^2^,(C_n_)^5^, and(C_n_)^7^. Only modification with a C_10_ fatty acid was examined for Positions 1, 4, and 8, whereas introduction of fatty acid chains of varying lengths (C_4_ to C_14_) was examined for Positions 2, 5, and 7. Generally it was found that the positioning of the fatty acid seemed to be important for the antimicrobial activity. When the fatty acid was inserted in Position 5 or 7, the activity against several bacterial species were enhanced significantly as compared to that of other subtypes. Overall, the best antimicrobial activity was obtained when C_8_–C_12_ fatty acids were inserted at Positions 5 and 7. For shorter and longer lipid tails, the antimicrobial activity was lowered considerably or even completely lost. However, it was not possible to enhance the antimicrobial activity as compared to that of the original nonacylated S3(B).

## Figures and Tables

**Table 1:** Minimum inhibitory concentration values (μg/mL).

Nr	ID	Peptide	EC ^a^	SA ^b^	AB ^c^	PA ^d^	KP ^e^	%H ^f^	%B ^g^
S3(B)		c(Dab-Nal-Arg-Dab-Bip-O2Oc-Nal-Dab-Asn)	16	8	16	8	>64	77	71
**1**	**(C_10_)^1^**	c(**Dab(C_10_)**-Nal-Arg-Dab-Bip-O2Oc-Nal-Dab-Asn)	>64	8	8	64	64	100	84
**2**	**(C_10_)^2^**	c(Dab-**Dab(C_10_)**-Arg-Dab-Bip-O2Oc-Nal-Dab-Asn)	32	4	16	8	>64	100	76
**3**	**(C_12_)^2^**	c(Dab-**Dab(C_12_)**-Arg-Dab-Bip-O2Oc-Nal-Dab-Asn)	64	16	64	32	>64	100	79
**4**	**(C_14_)^2^**	c(Dab-**Dab(C_14_)**-Arg-Dab-Bip-O2Oc-Nal-Dab-Asn)	>64	64	>64	>64	>64	100	84
**5**	**(C_10_)^4^**	c(Dab-Nal-Arg-**Dab(C_10_)**-Bip-O2Oc-Nal-Dab-Asn)	64	8	16	>64	64	100	85
**6**	**(C_4_)^5^**	c(Dab-Nal-Arg-Dab-**Dab(C_4_)**-O2Oc-Nal-Dab-Asn)	64	>64	>64	32	>64	5	62
**7**	**(C_6_)^5^**	c(Dab-Nal-Arg-Dab-**Dab(C_6_)**-O2Oc-Nal-Dab-Asn)	32	32	64	16	>64	13	65
**8**	**(C_8_)^5^**	c(Dab-Nal-Arg-Dab-**Dab(C_8_)**-O2Oc-Nal-Dab-Asn)	16	8	16	8	>64	42	69
**9**	**(C_10_)^5^**	c(Dab-Nal-Arg-Dab-**Dab(C_10_)**-O2Oc-Nal-Dab-Asn)	16	4	8	4	>64	100	74
**10**	**(C_12_)^5^**	c(Dab-Nal-Arg-Dab-**Dab(C_12_)**-O2Oc-Nal-Dab-Asn)	32	4	16	8	>64	100	78
**11**	**(C_14_)^5^**	c(Dab-Nal-Arg-Dab-**Dab(C_14_)**-O2Oc-Nal-Dab-Asn)	32	16	>64	64	>64	100	83
**12**	**(C_4_)^7^**	c(Dab-Nal-Arg-Dab-Bip-O2Oc-**Dab(C_4_)**-Dab-Asn)	64	64	64	64	>64	4	64
**13**	**(C_6_)^7^**	c(Dab-Nal-Arg-Dab-Bip-O2Oc-**Dab(C_6_)**-Dab-Asn)	32	16	64	16	>64	8	69
**14**	**(C_8_)^7^**	c(Dab-Nal-Arg-Dab-Bip-O2Oc-**Dab(C_8_)**-Dab-Asn)	16	8	16	8	>64	56	71
**15**	**(C_10_)^7^**	c(Dab-Nal-Arg-Dab-Bip-O2Oc-**Dab(C_10_)**-Dab-Asn)	16	4	8	8	>64	100	74
**16**	**(C_12_)^7^**	c(Dab-Nal-Arg-Dab-Bip-O2Oc-**Dab(C_12_)**-Dab-Asn)	16	4	16	8	>64	96	79
**17**	**(C_14_)^7^**	c(Dab-Nal-Arg-Dab-Bip-O2Oc-**Dab(C_14_)**-Dab-Asn)	64	16	>64	64	>64	98	84
**18**	**(C_10_)^8^**	c(Dab-Nal-Arg-Dab-Bip-O2Oc-Nal-**Dab(C_10_)**-Asn)	64	4	16	>64	>64	100	84
**19**	**Ref**	Colistin	0.25	N/A	0.25	0.25	0.5	N/A	N/A
**20**	**Ref**	Vancomycin	N/A	0.5	N/A	N/A	N/A	N/A	N/A

^a^*E. coli* ATCC 29,522 (EC), ^b^ *S. aureus* ATCC 29,213 (SA)), ^c^ *A. baumannii* ATCC 19,606 (AB), ^d^ *P. aeruginosa* ATCC 27,853 (PA), ^e^ *K. pneumoniae* (KP), ^f^ percent hemolytic activity against red blood cells at 150 μM. ^g^ %B at peak of elution in analytical HPLC. N/A: not applicable.

## Supplementary Materials

The following are available online at https://www.mdpi.com/1422-0067/21/16/5829/s1. Figure S1: Structure of cyclic lipopeptides; Table S1: Peptide mass, HPLC retention time and purity; Table S2: Overview of the analytical data obtained by MALDI-TOF-MS and the peptide purity after purification; Table S3: Analytical chromatograms before and after purification III: MALDI-TOF-MS spectra of all purified peptides.

## Abbreviations

ACN	Acetonitrile
Bip	l-biphenylalanine
BSA	Bovine Serum Albumin
CFU	Colony-forming unit
Dab	l-2,4-diaminobutyric acid
DCM	Dichloromethane
DIEA	Disopropylethylamine
Dmb	2,4-Dimethoxybenzyl
DMF	Dimethylformamide
DMTMM.BF_4_	4-(4,6-dimethoxy-1,3,5-triazin-2-yl)-4-methylmorpholinium tetrafluoroborate
DTT	Dithiothreitol
EUCAST	European Committee on Antimicrobial Susceptibility Testing
Fmoc	9-fluorenylmethoxycarbonyl
HA	Hemolytic activity
HATU	1-[*Bis*(dimethylamino)methylene]-1*H*-1,2,3-triazolo[4,5-b]pyridinium-3-oxid hexafluoro-phosphate, *N*-[(Dimethylamino)-1*H*-1,2,3-triazolo-[4,5-b]pyridin-1-ylmethylene]-*N*-methylmethan-aminium hexa-fluorophosphate *N*-oxide)
HOAt	1-Hydroxy-7-azabenzotriazole
IvDde	1-(4,4-dimethyl-2,6-dioxocyclohex-1-ylidene)-3-methylbutyl
LPS	Lipopolysaccharide
MALDI-TOF MS	Matrix-assisted linear desorption Time-Of-Flight Mass Spectrometry
MHB	Mueller-Hinton broth
MIC	Minimum Inhibitory Concentration
Nal	3-(2-Naphthyl)-l-alanine
O_2_Oc	8-amino-3,6-dioxaoctanoic acid
PBS	Phosphate-buffered saline
RAM	Rink amide
RBC	Red blood cell
RP-HPLC	Reverse Phase Analytical High Performance Liquid Chromatography
rpm	Rotations per minute
SPPS	Solid-Phase Peptide Synthesis
TFA	Trifluoroacetic acid
TIS	Triisopropylsilane
