# Supplementary material for: Novel Cyclic Lipopeptide Antibiotics: Effects of Acyl Chain Length and Position
Source: Int J Mol Sci. 2020 Aug 13;21(16):5829. doi: 10.3390/ijms21165829 (PMC7461568; doi:10.3390/ijms21165829)
Supplement: Supplementary file 1 [file ijms-21-05829-s001.pdf]

International Journal of
Molecular Sciences

MDPI

Supplementary Materials

Novel Cyclic Lipopeptide Antibiotics: Effects of Acyl Chain Length and Position

Signe Kaustrup Jensen [1], Thomas T. Thomsen [2,3], Alberto Oddo [1,†], Henrik Franzyk [1], Anders Løbner-Olesen [3] and Paul R. Hansen [1,*]

[1] Department of Drug Design and Pharmacology, Faculty of Health and Medical Sciences, University of Copenhagen, Universitetsparken 2, 2100 Copenhagen, Denmark; signekaustrupjensen@outlook.com (S.K.J.); albi.oddo@gmail.com (A.O.); henrik.franzyk@sund.ku.dk (H.F.)

[2] Department of Clinical Microbiology, Rigshospitalet, Henrik HarpestrengsVej 4A, 2100 Copenhagen. Denmark; thomas.thomsen@bio.ku.dk

[3] Department of Biology, Section for functional Genomics, University of Copenhagen, Ole MaaløesVej 5, 2200 Copenhagen, Denmark; lobner@bio.ku.dk

* Correspondence: prh@sund.ku.dk; Tel.: +45-35336625

† Current address: Novo Nordisk A/S, Krogshøjvej 44, 2820 Bagsværd, Denmark

Content:

Figure S1: Structure of cyclic peptides

Table S1. Peptide mass, HPLC retention time and purity.

Table S2.: Overview of the analytical data obtained by MALDI-TOF-MS and the peptide purity after purification

Table S3.: Analytical chromatograms before and after purification III: MALDI-TOF-MS spectra of all purified peptides

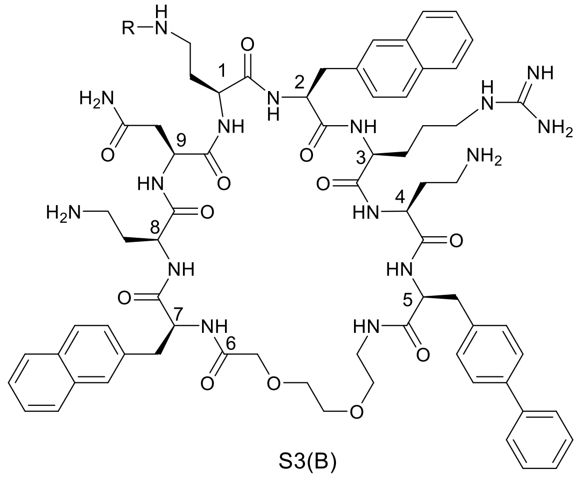

S3(B)

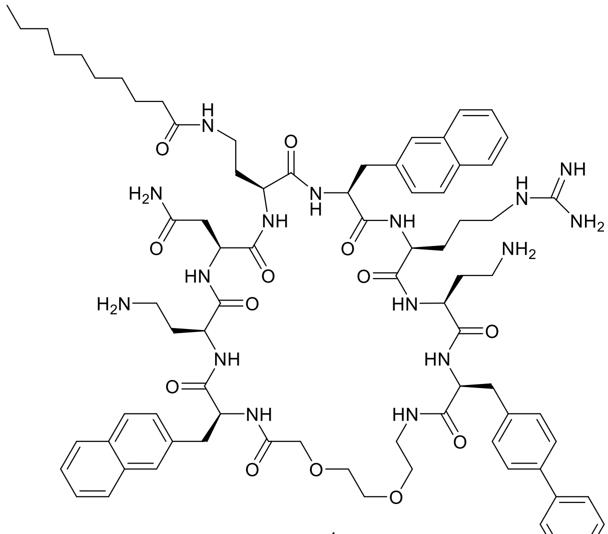

1

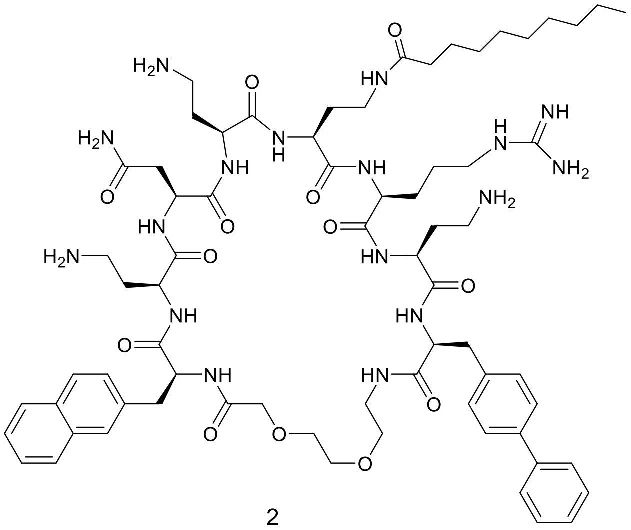

2

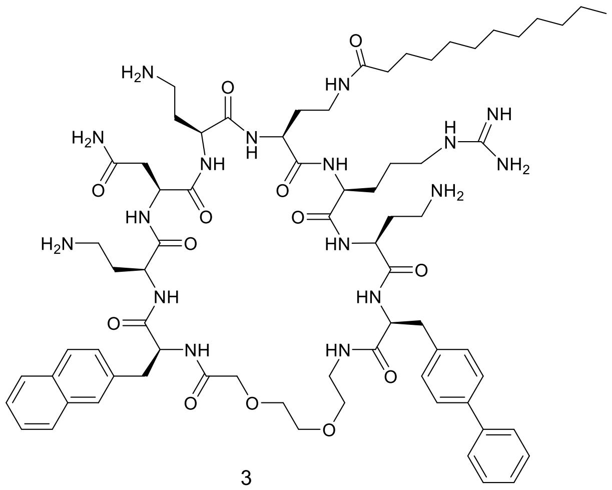

3

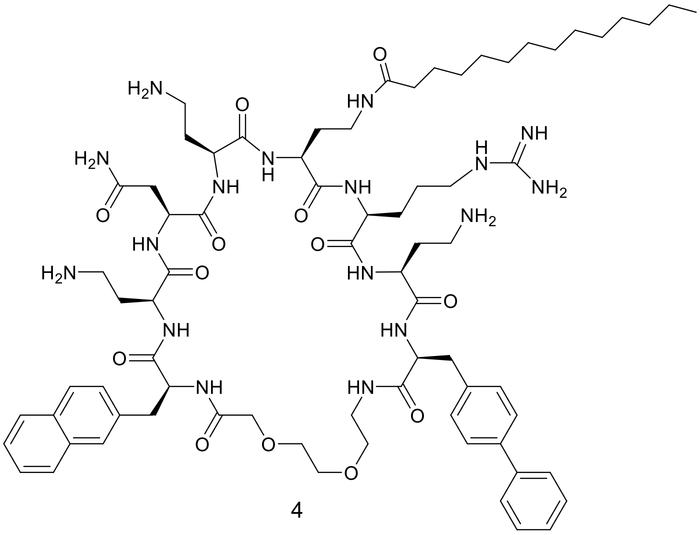

4

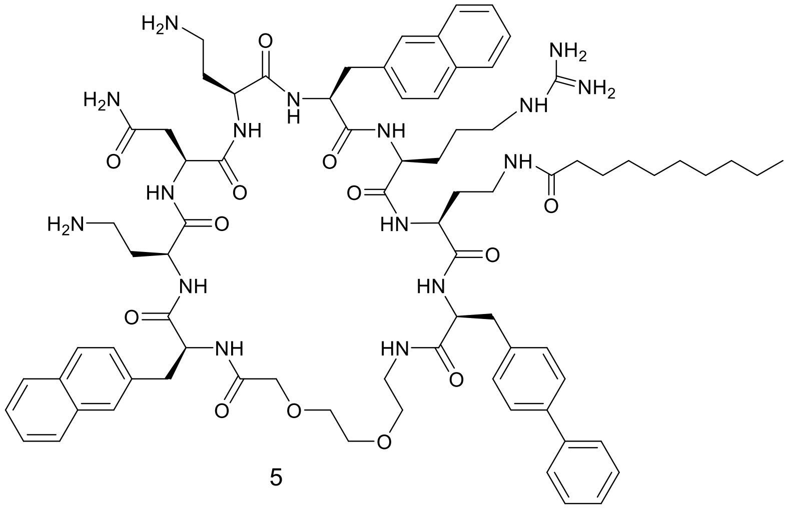

5

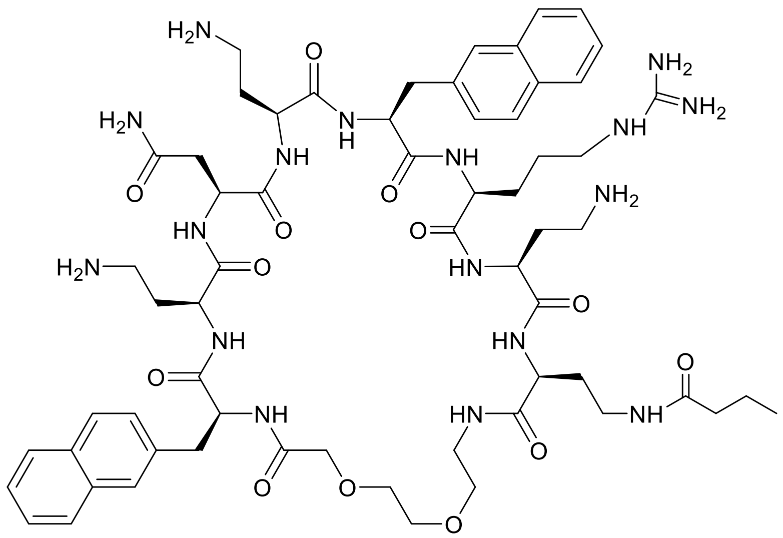

6

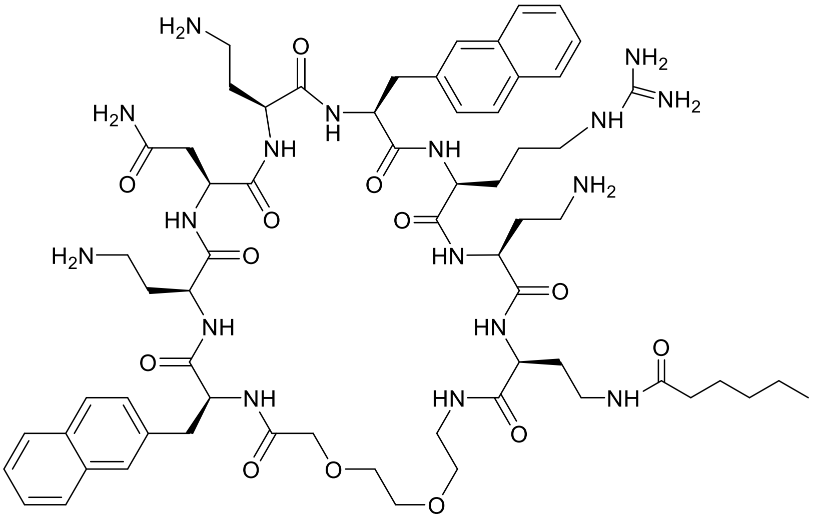

7

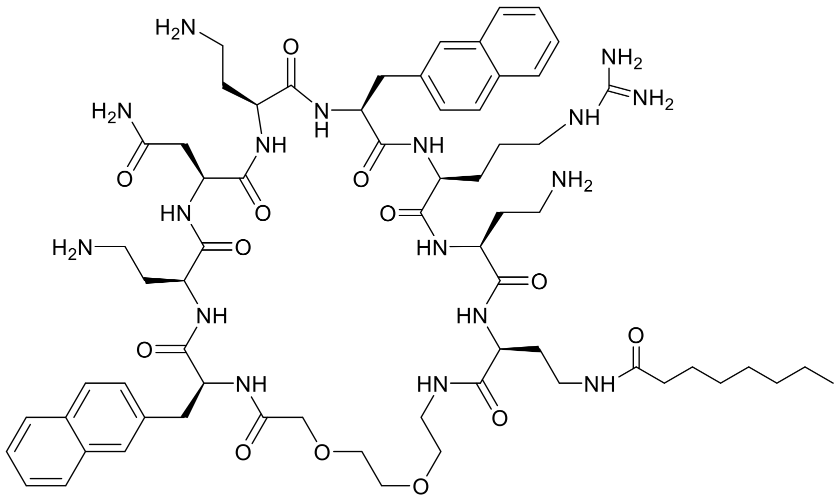

8

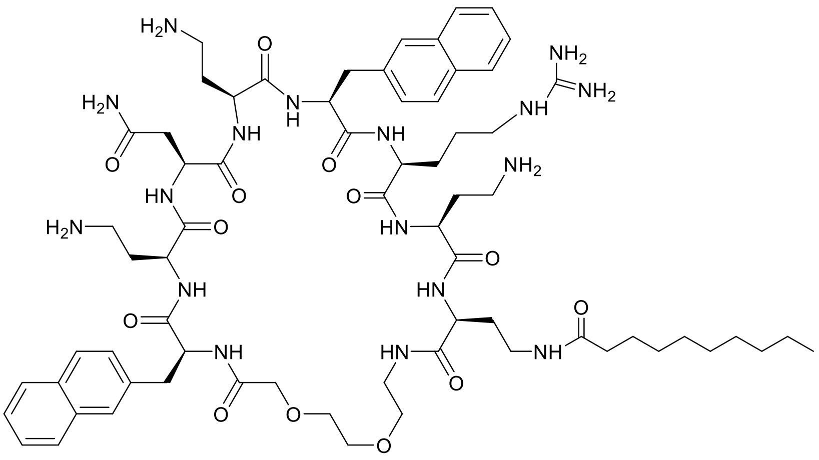

9

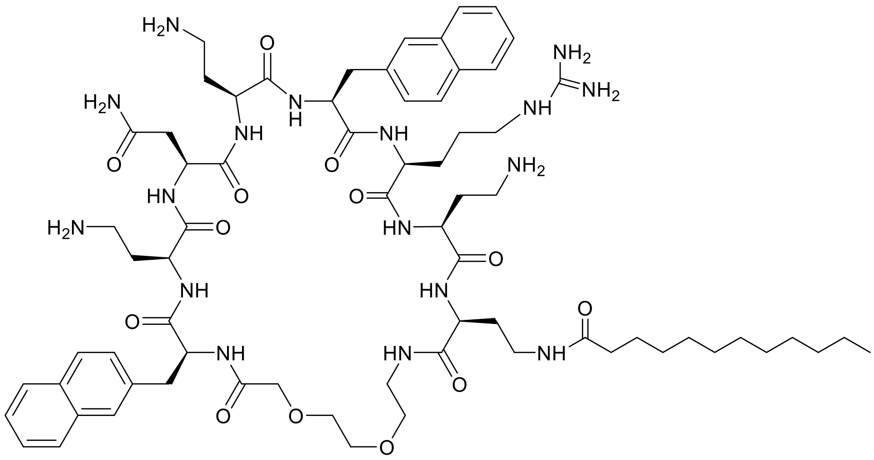

10

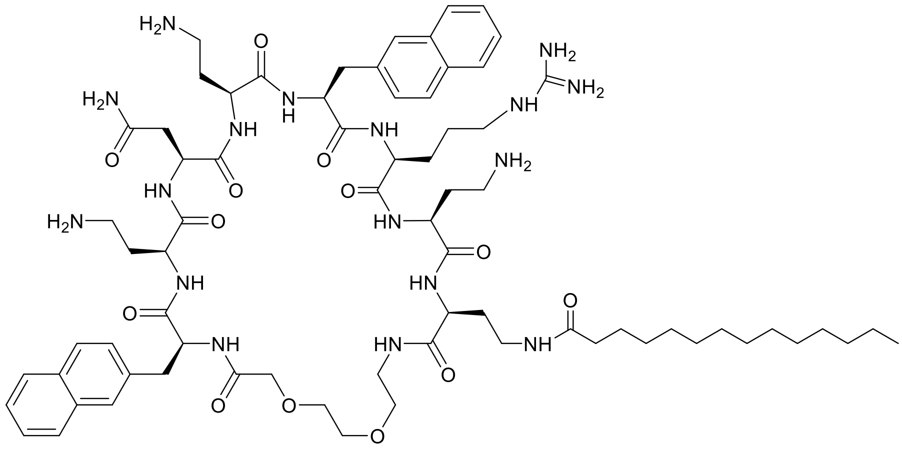

11

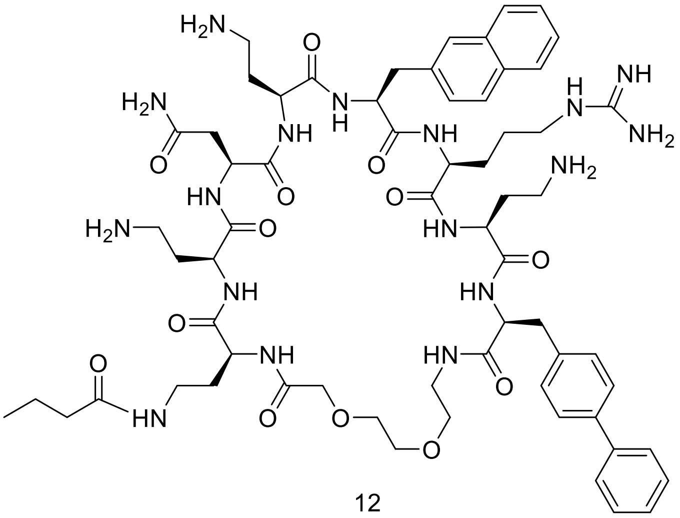

12

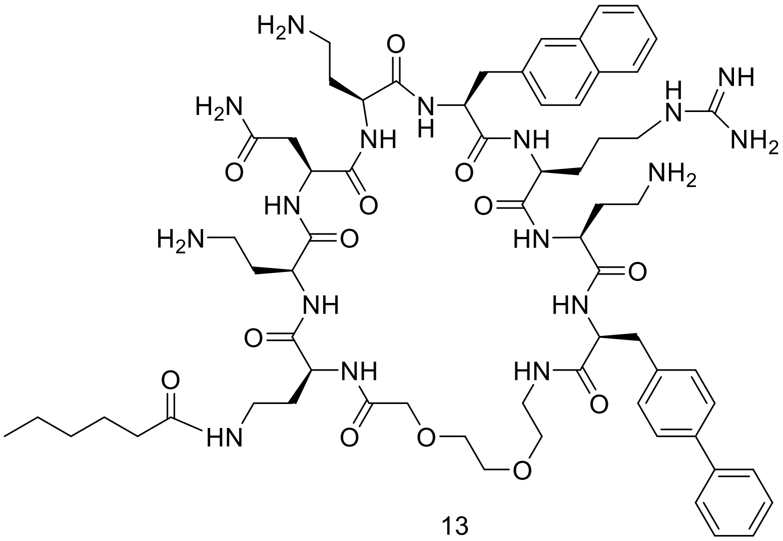

13

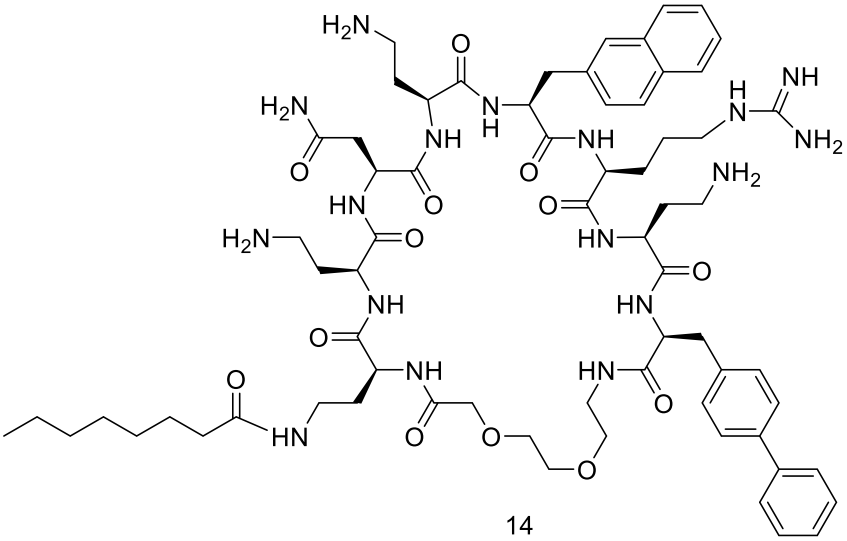

14

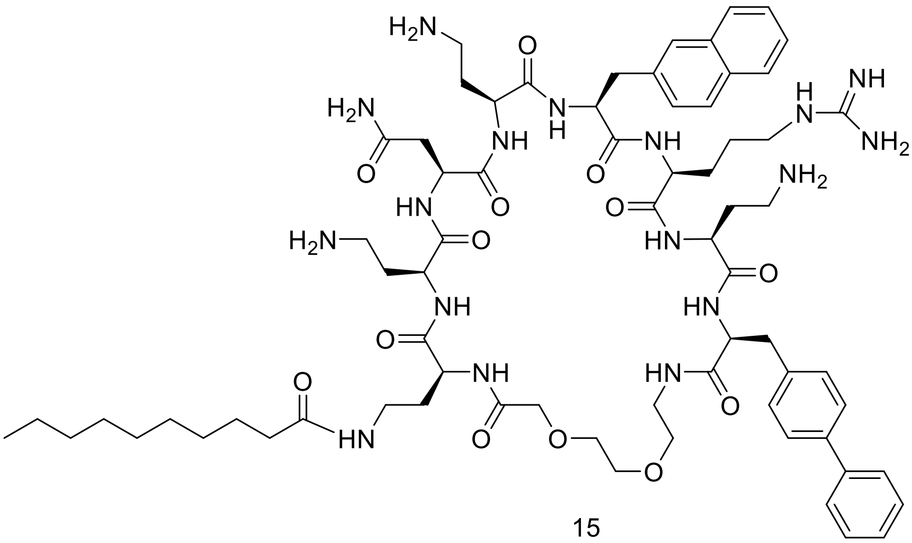

15

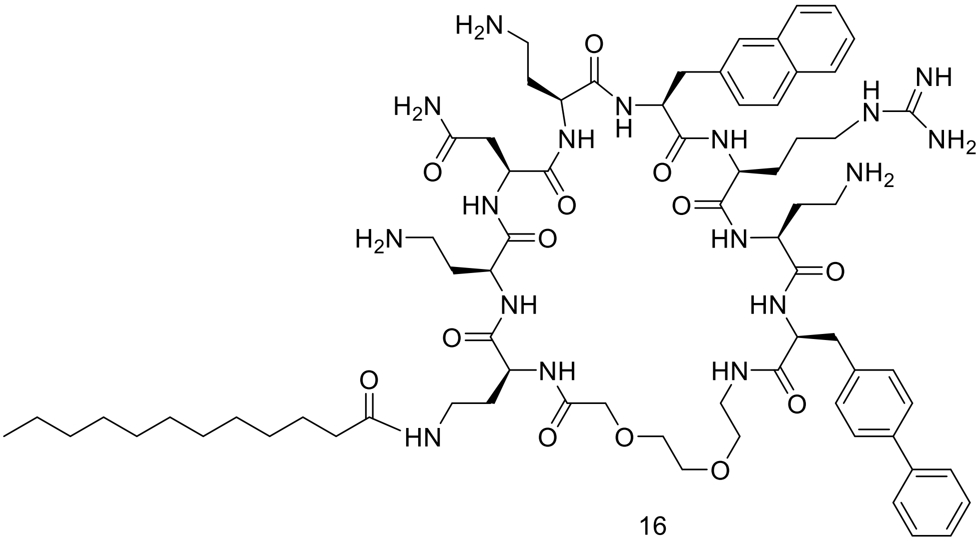

16

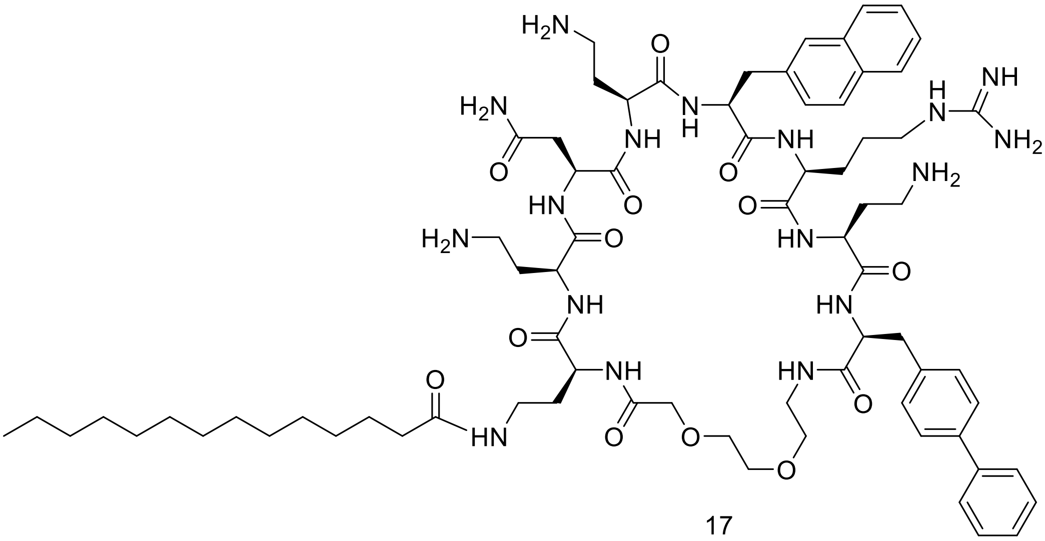

17

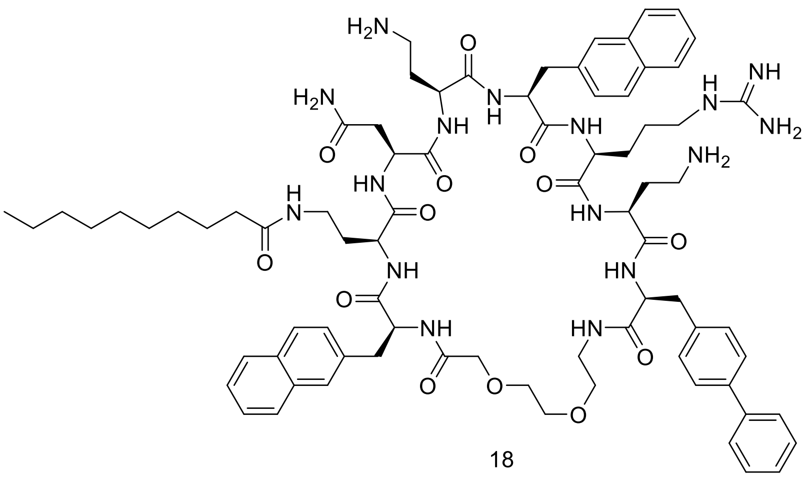

18

Figure S1. Structure of cyclic peptides. (**S3(B)**) and (**1–18**) are cyclic lipopeptides

Table S1. Peptide mass, HPLC retention time and purity.

Peptide		Mw [a]	[MH+] [b]	t_R (min)[c]	Purity
S3(B)	S3(B)	1333.68	1334.37	14.1	99.5%
1	(C_{10})[1]	1487.82	1488.98	16.7	98.2%
2	(C_{10})[2]	1390.70	1391.53	15.1	98.6%
3	(C_{12})[2]	1418.75	1419.48	15.8	98.6%
4	(C_{14})[2]	1446.81	1447.61	16.7	97.6%
5	(C_{10})[4]	1487.82	1489.07	17.0	98.9%
6	(C_4)[5]	1280.50	1281.63	12.3	95.1%
7	(C_6)[5]	1308.55	1309.66	13.0	98.1%
8	(C_8)[5]	1336.61	1337.77	13.8	100%
9	(C_{10})[5]	1364.66	1365.58	14.8	97.4%
10	(C_{12})[5]	1392.72	1393.79	15.6	98.0%
11	(C_{14})[5]	1420.77	1421.53	16.6	98.8%
12	(C_4)[7]	1306.54	1307.75	12.7	95.1%
13	(C_6)[7]	1334.59	1335.28	13.7	99.1%
14	(C_8)[7]	1362.65	1363.68	14.1	96.0%
15	(C_{10})[7]	1390.70	1391.43	14.8	98.8%
16	(C_{12})[7]	1418.75	1419.60	15.8	97.3%
17	(C_{14})[7]	1446.81	1447.74	16.8	98.4%
18	(C_{10})[8]	1487.82	1488.89	16.8	99.5%

a Molecular weight of the peptides, g/mol; b Mass-to-charge value; c Retention time obtained by RP-HPLC.

Table S2. Overview of the analytical data obtained by MALDI-TOF-MS and the peptide purity after purification.

Analytical HPLC chromatograms	Cyclic Lipopeptide	Analytical HPLC chromatograms
$(C_{10})^1$	Crude<br>t_R: 16.7 min	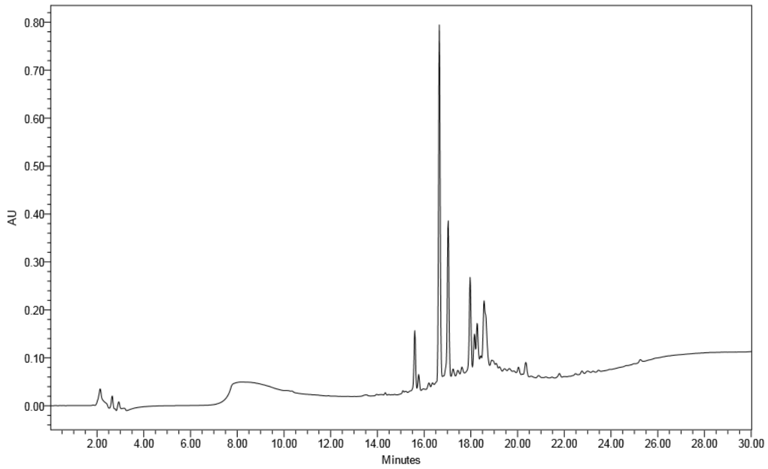
$(C_{10})^1$	Purified<br>t_R: 16.7 min<br>Purity: 98.2%	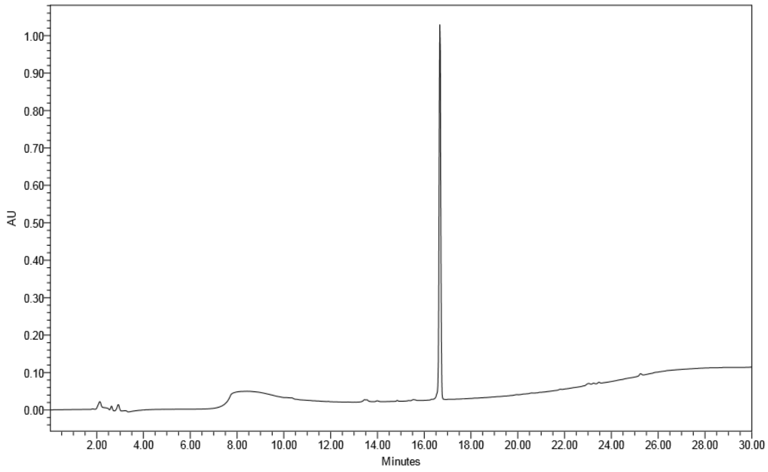
$(C_{10})^2$	Crude<br>t_R: 18.6 min<br>(method *Pep1*)	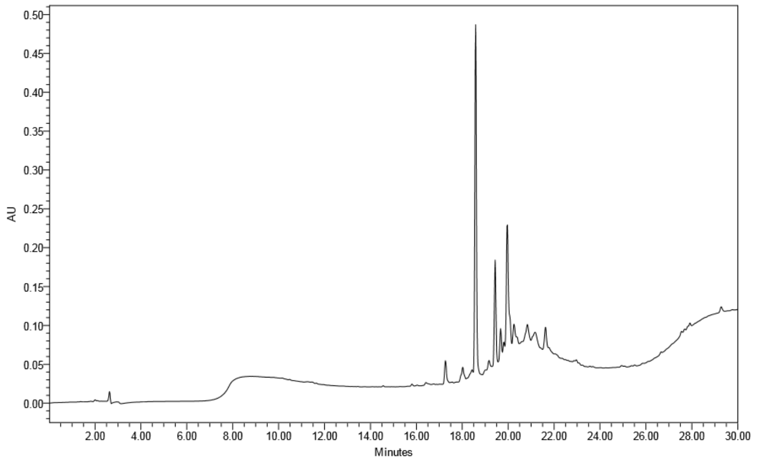

$(C_{10})^2$	<u>Purified</u><br>t_R: 18.1 min<br>Purity: 98.6%	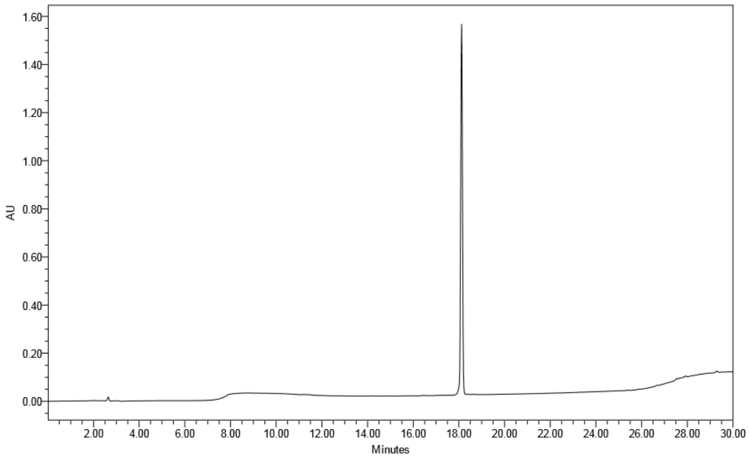
$(C_{12})^2$	<u>Crude</u><br>t_R: 15.7 min	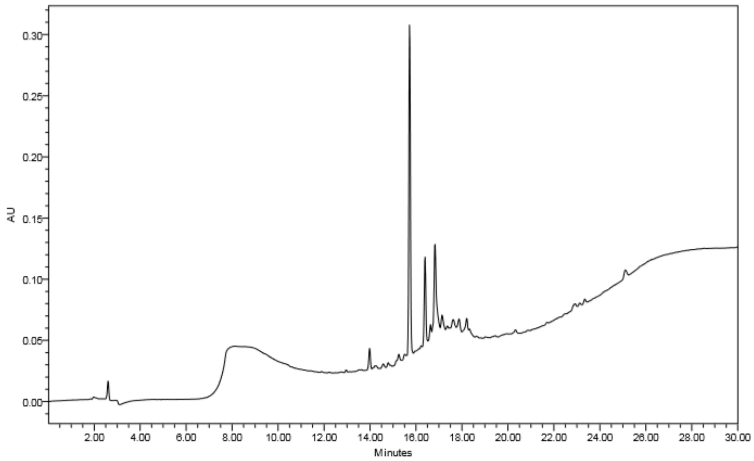
$(C_{12})^2$	<u>Purified</u><br>t_R: 15.8 min<br>Purity: 98.6%	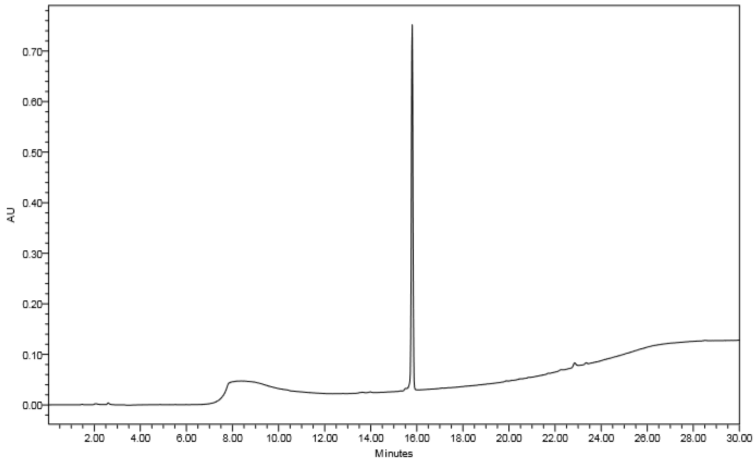

$(C_{14})^2$	<u>Crude</u><br>t_R: 16.6 min	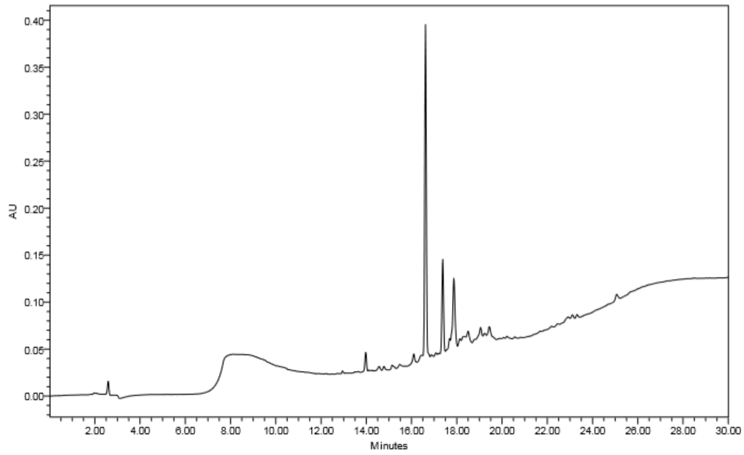
$(C_{14})^2$	<u>Purified</u><br>t_R: 16.7 min<br>Purity: 97.6%	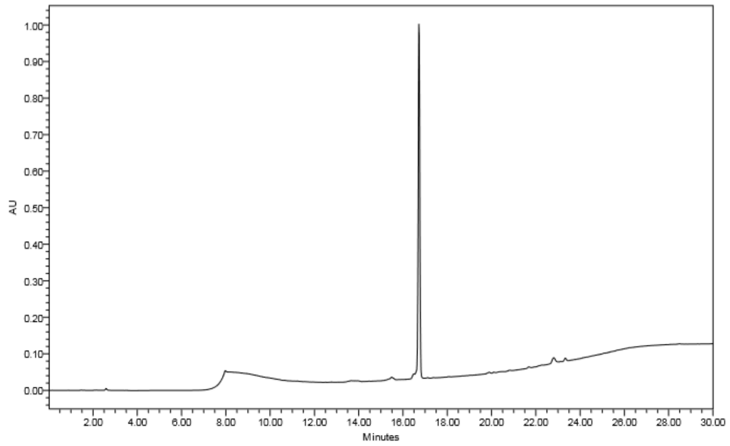
$(C_{10})^4$	<u>Crude</u><br>t_R: 17.0 min	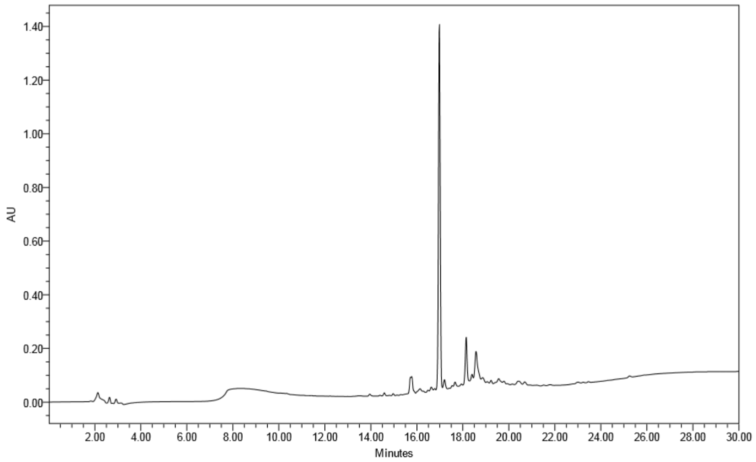

$(C_{10})^4$	<u>Purified</u><br>t_R: 17.0 min<br>Purity: 98.9%	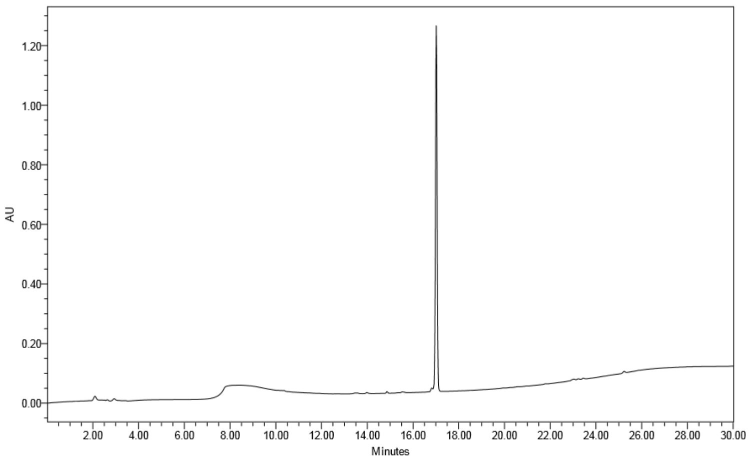
$(C_4)^5$	<u>Crude</u><br>t_R: 12.3 min	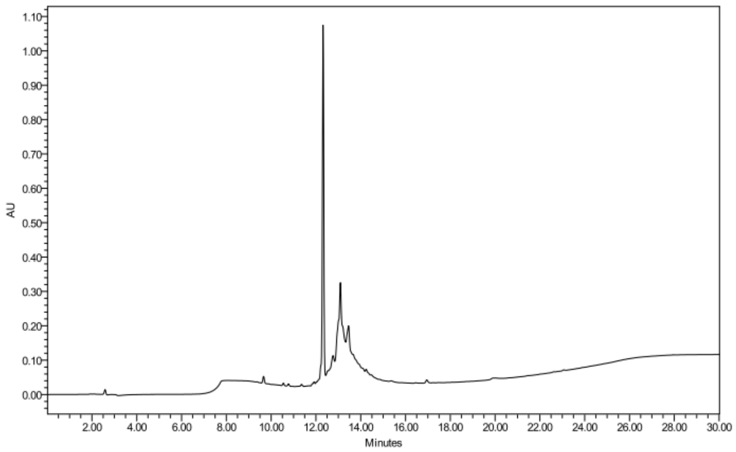
$(C_4)^5$	<u>Purified</u><br>t_R: 12.3 min<br>Purity: 95.1%	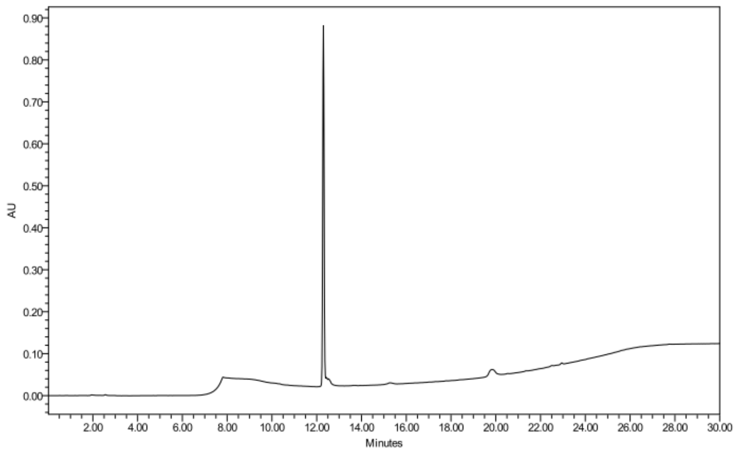

$(C_6)^5$	<u>Crude</u><br>t_R: 13.0 min	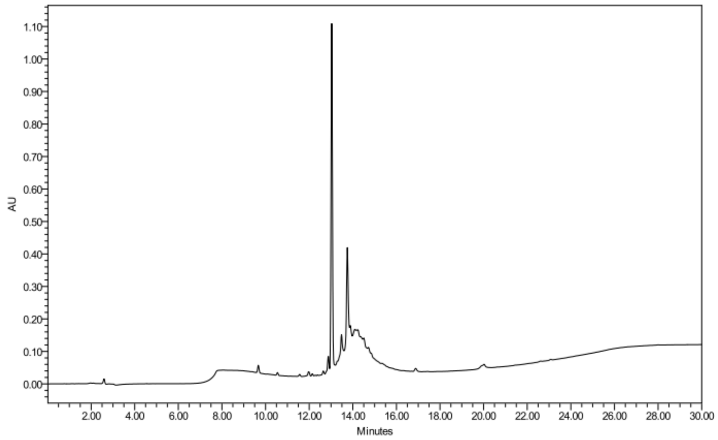
$(C_6)^5$	<u>Purified</u><br>t_R: 13.0 min<br>Purity: 98.1%	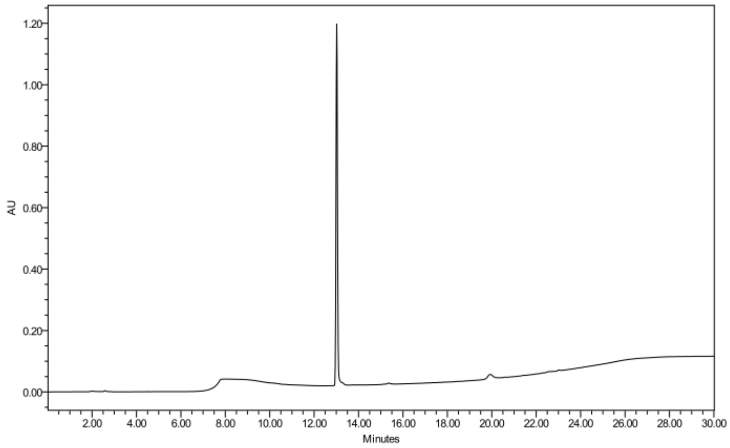
$(C_8)^5$	<u>Crude</u><br>t_R: 13.8 min	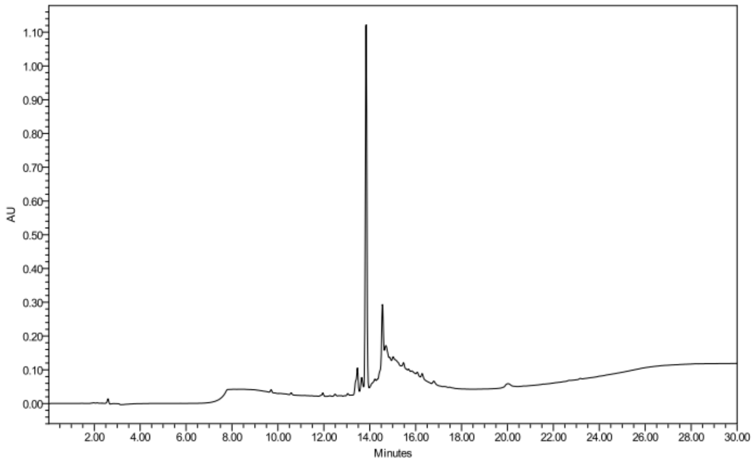

$(C_8)^5$	Purified<br>t_R: 13.8 min<br>Purity: 100%	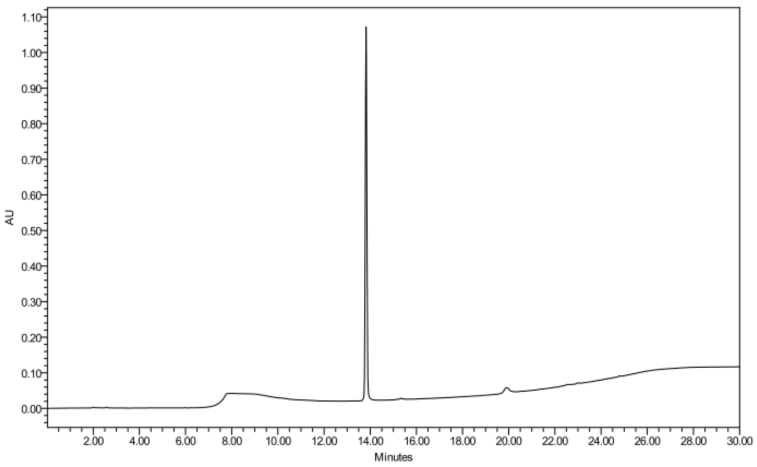
$(C_{10})^5$	Crude<br>t_R: 18.2 min<br>(method<br>*Pep1*)	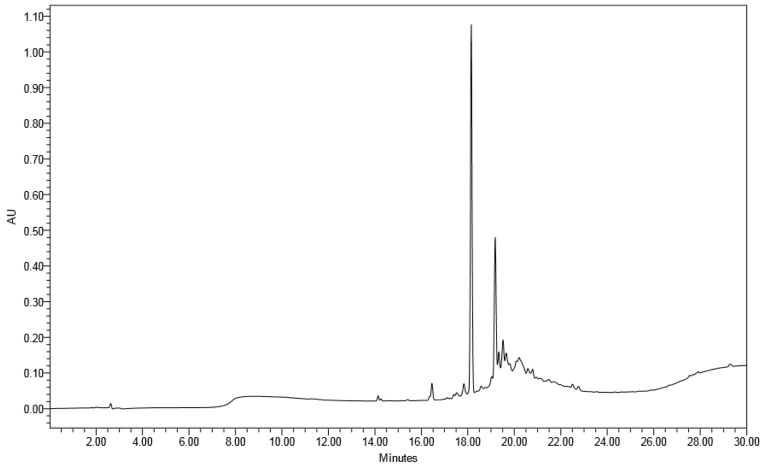
$(C_{10})^5$	Purified<br>t_R:18.6 min<br>(method<br>*Pep1*(shown)<br>)/14.8 min<br>(method<br>*0_100 %B*)<br>Purity: 97.4%	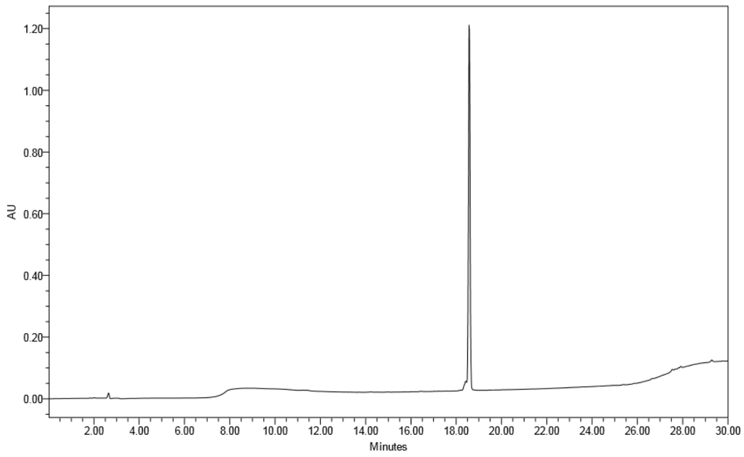

$(C_{12})^5$	<u>Crude</u><br>t_R: 15.5 min	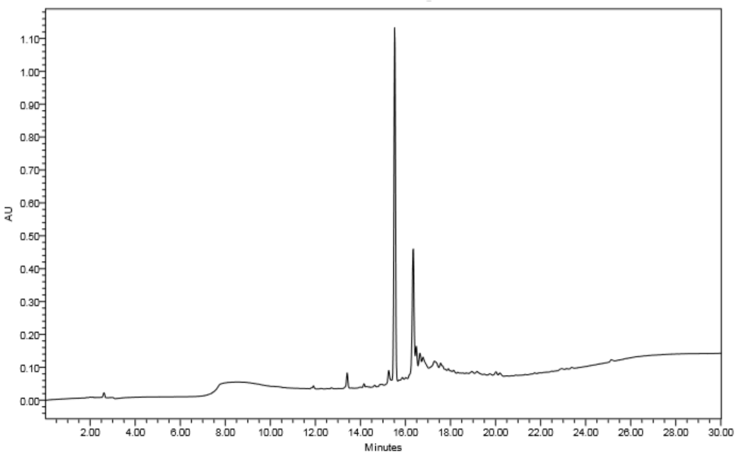
$(C_{12})^5$	<u>Purified</u><br>t_R: 15.6 min<br>Purity: 98.0%	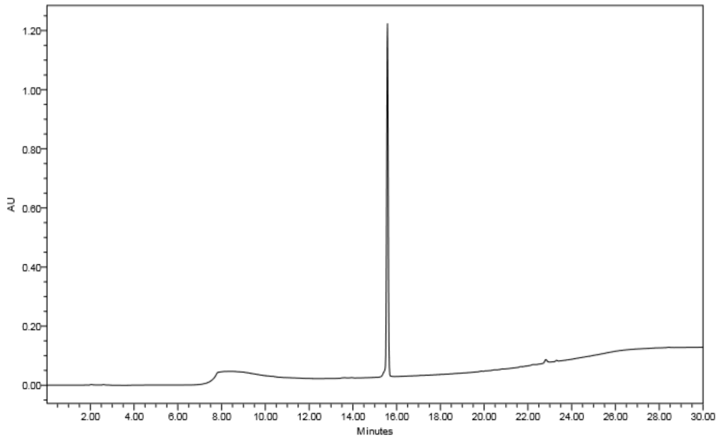
$(C_{14})^5$	<u>Crude</u><br>t_R: 16.5 min	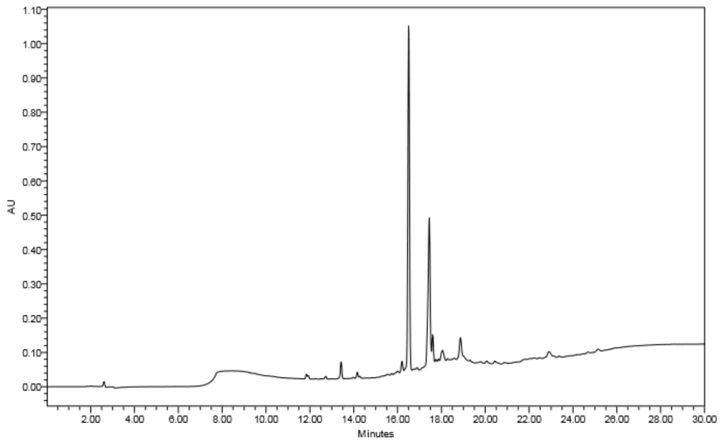

$(C_{14})^5$	Purified<br>t_R: 16.6 min<br>Purity: 98.8%	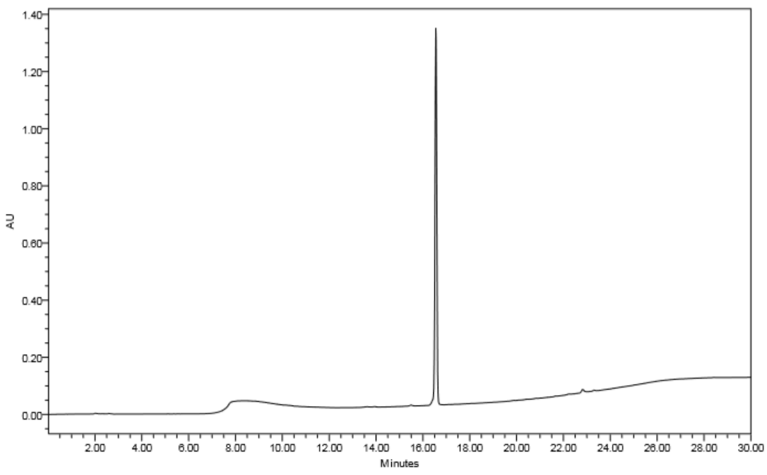
$(C_4)^7$	Crude<br>t_R: 12.7 min	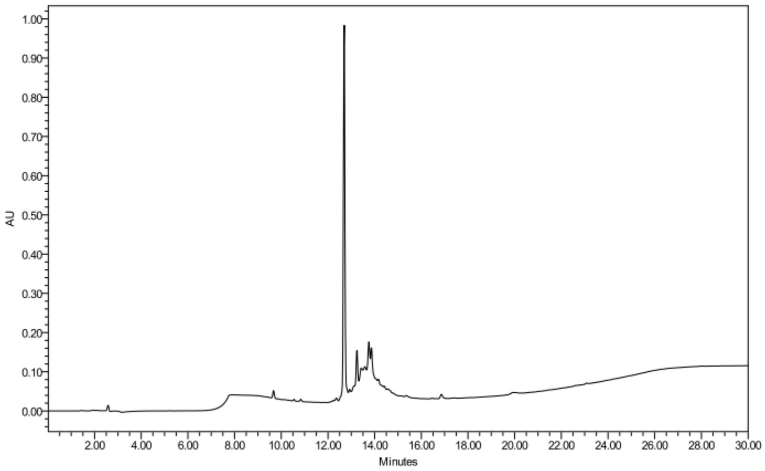
$(C_4)^7$	Purified<br>t_R: 12.7 min<br>Purity: 95.1%	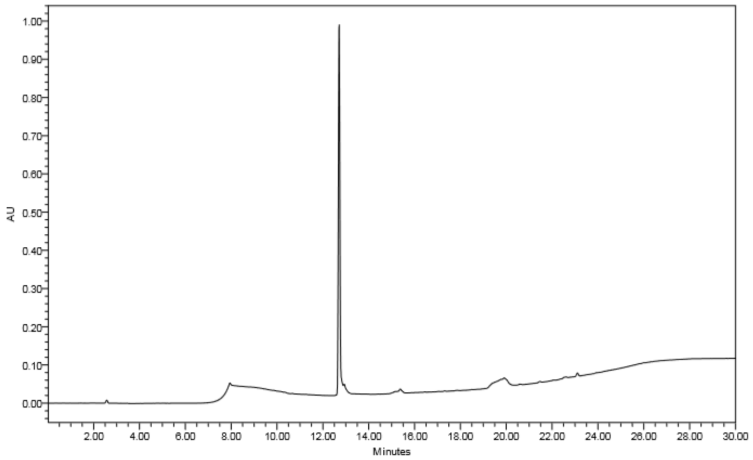

$(C_6)^7$

<u>Crude</u>
t_R: 13.4 min

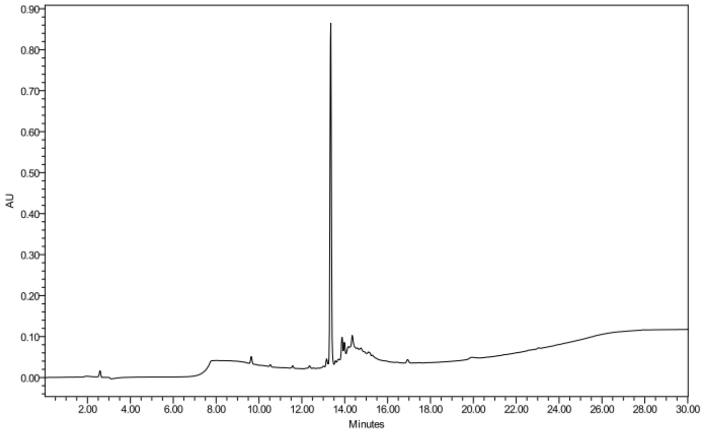

$(C_6)^7$

<u>Purified</u>
t_R: 13.7 min
Purity: 99.1%

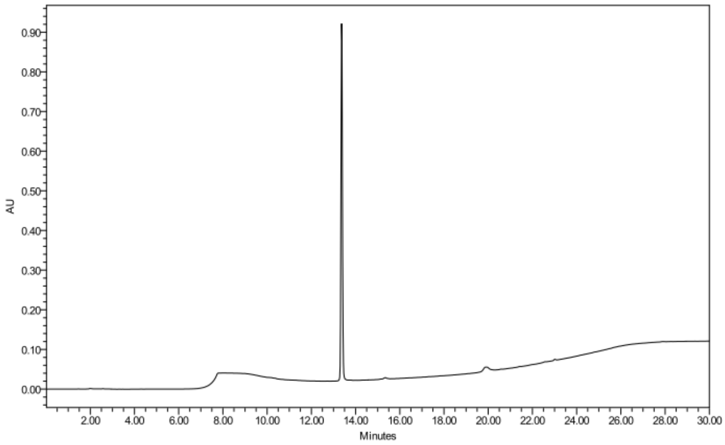

$(C_8)^7$

<u>Crude</u>
t_R: 14.1 min

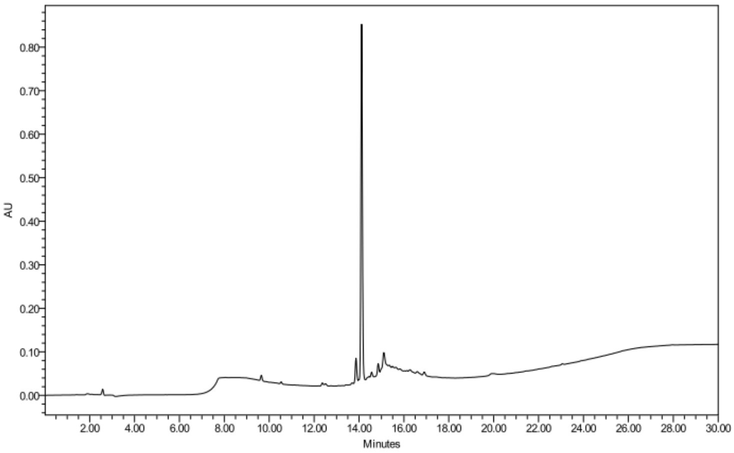

$(C_8)^7$

<u>Purified</u>
t_R: 14.1 min
Purity: 96.0%

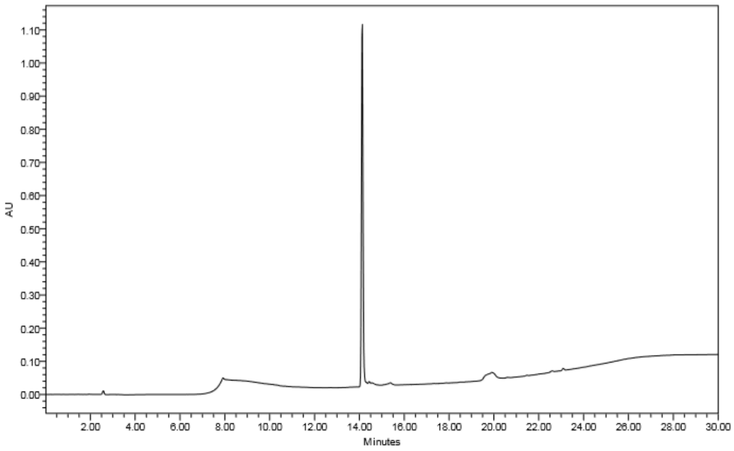

$(C_{10})^7$	<u>Crude</u><br>t_R: 14.8 min	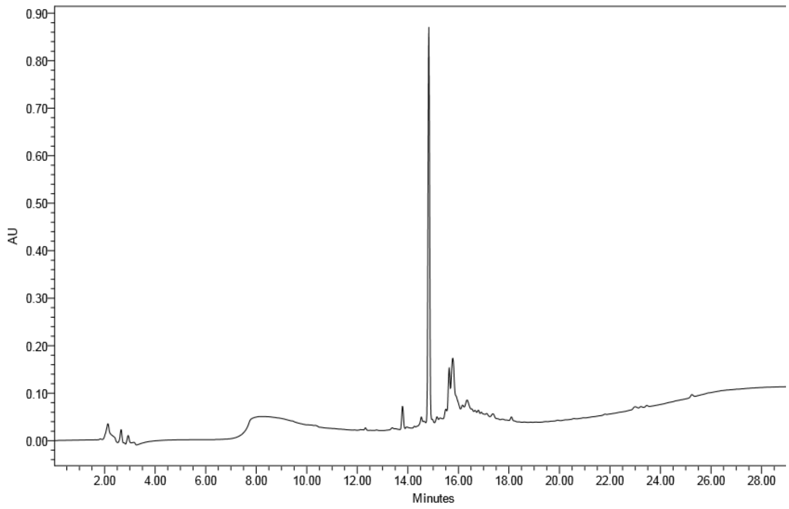
$(C_{10})^7$	<u>Purified</u><br>t_R: 14.8 min<br>Purity: 98.8%	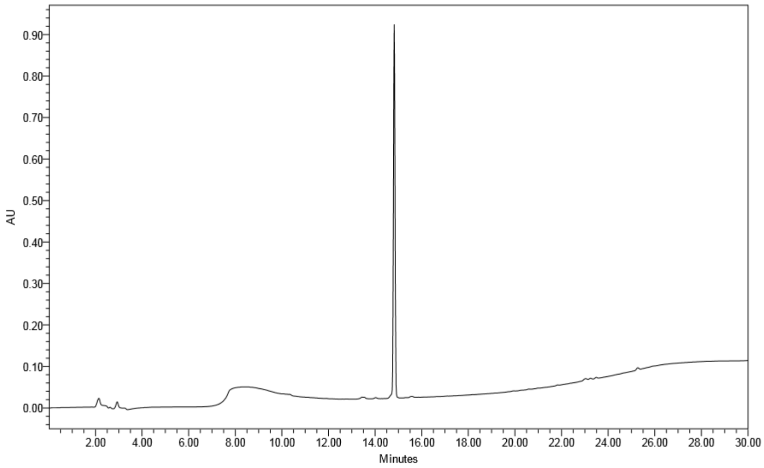
$(C_{12})^7$	<u>Crude</u><br>t_R: 15.8 min	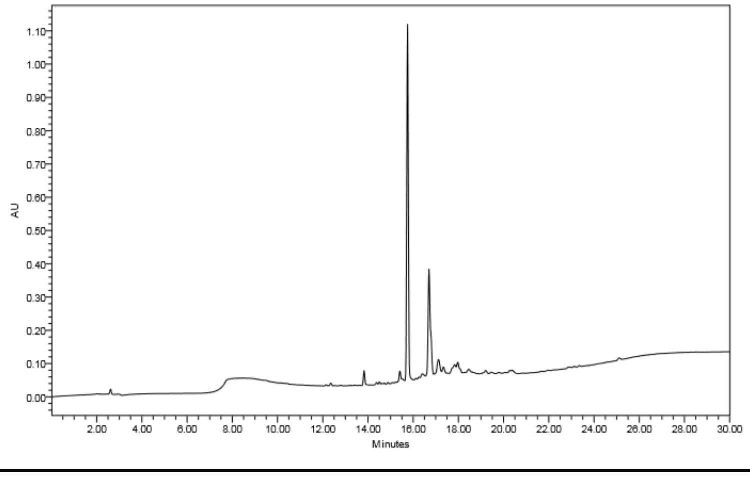

$(C_{12})^7$	<u>Purified</u><br>t_R: 15.8 min<br>Purity: 97.3%	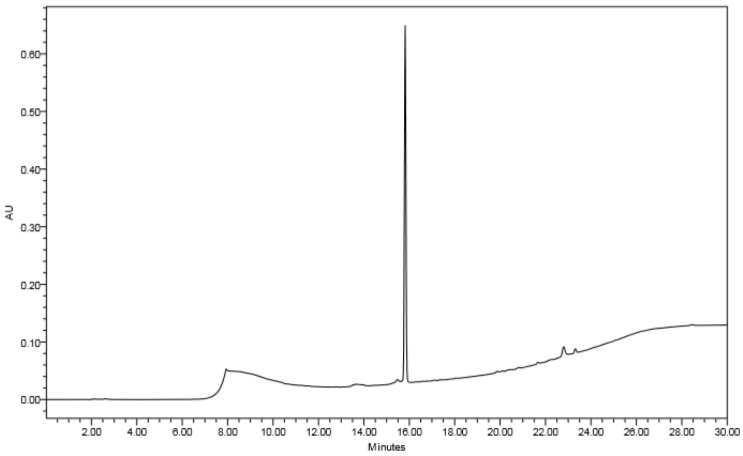
$(C_{14})^7$	<u>Crude</u><br>t_R: 16.7 min	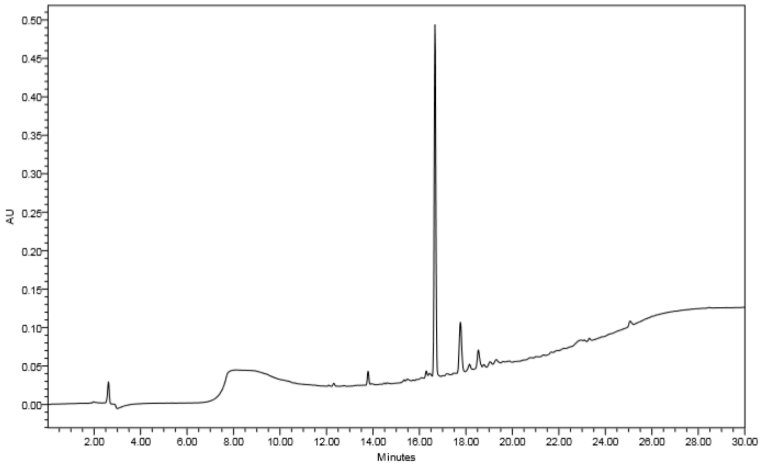
$(C_{14})^7$	<u>Purified</u><br>t_R: 16.8 min<br>Purity: 98.4%	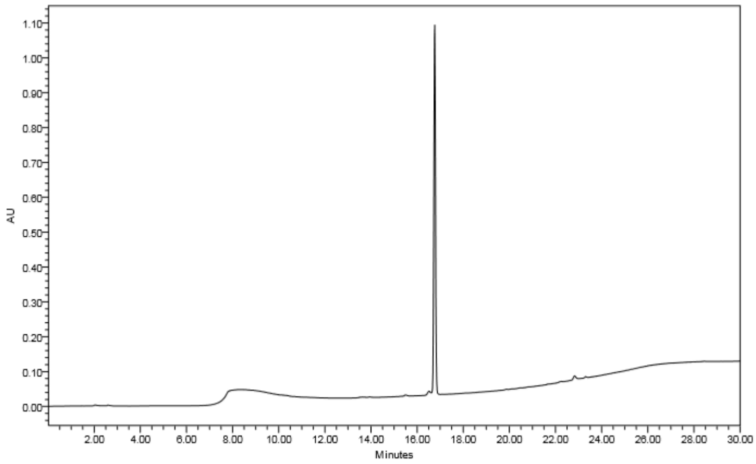

$(C_{10})^8$	<u>Crude</u><br>t_R:16.8 min	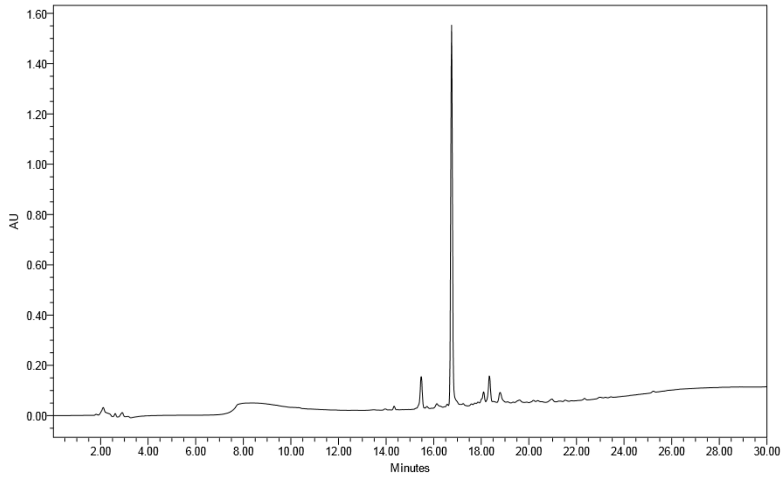
$(C_{10})^8$	<u>Purified</u><br>t_R: 16.8 min<br>Purity: 99.5%	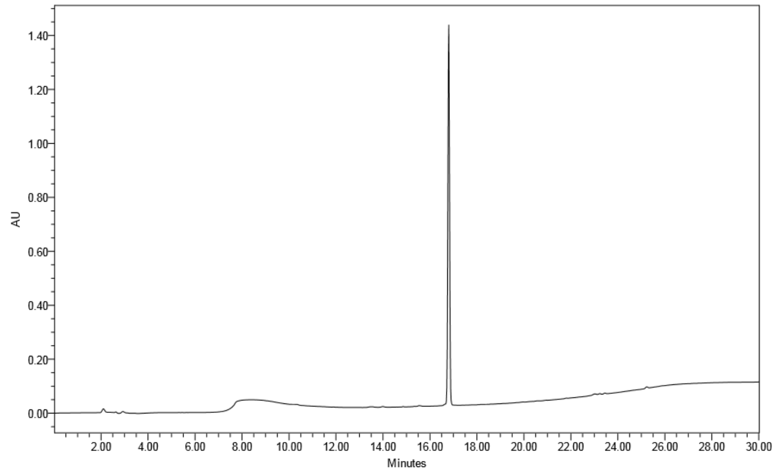
S3(B)	<u>Purified</u><br>t_R: 14.1 min<br>Purity: 99.5%	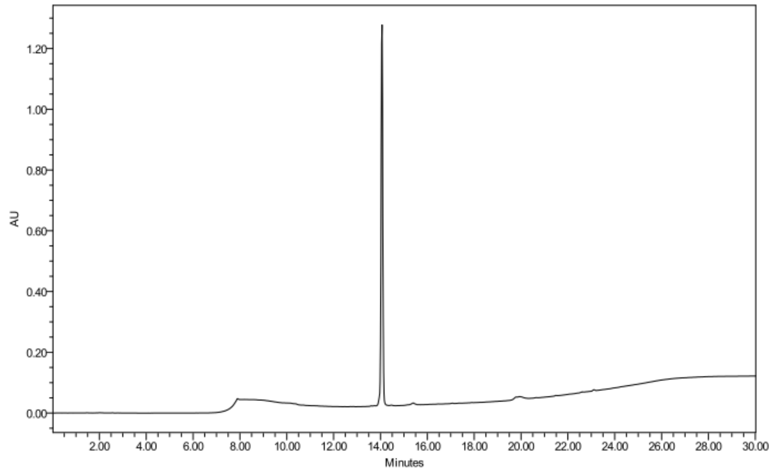

tR: retention time.

Table S3. Analytical chromatograms before and after purification III: MALDI-TOF-MS spectra of all purified peptides.

Cyclic Lipopeptide	MALDI-TOF-MS Spectra
FA1(C_{10})[1]<br>Mw: 1487.82 g/mol<br>[MH+]: 1488.981	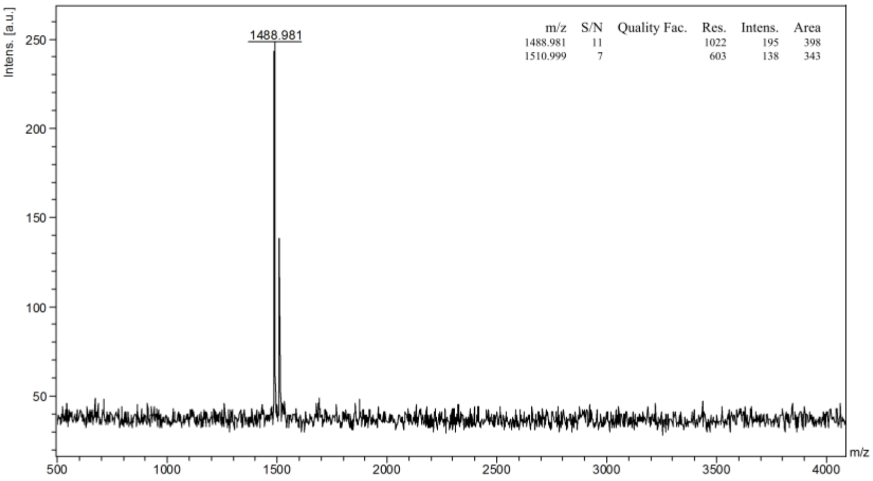
(C_{10})[2]<br>Mw: 1390.70 g/mol<br>[MH+]: 1391.532	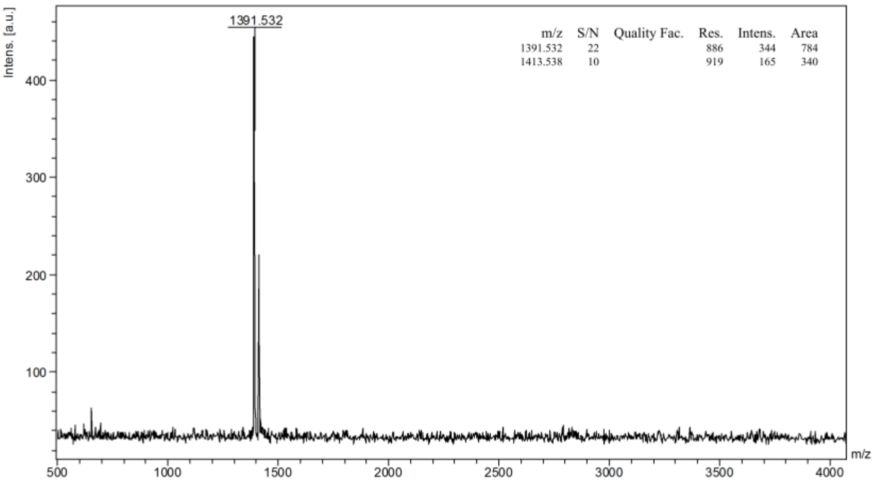
(C_{12})[2]<br>Mw: 1418.75 g/mol<br>[MH+]: 1419.477	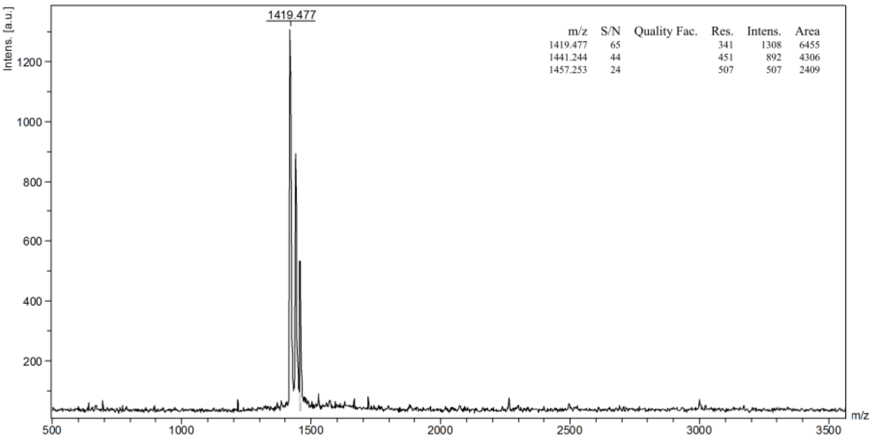

(C_{14})[2]
Mw: 1446.81 g/mol
[MH+]: 1447.610

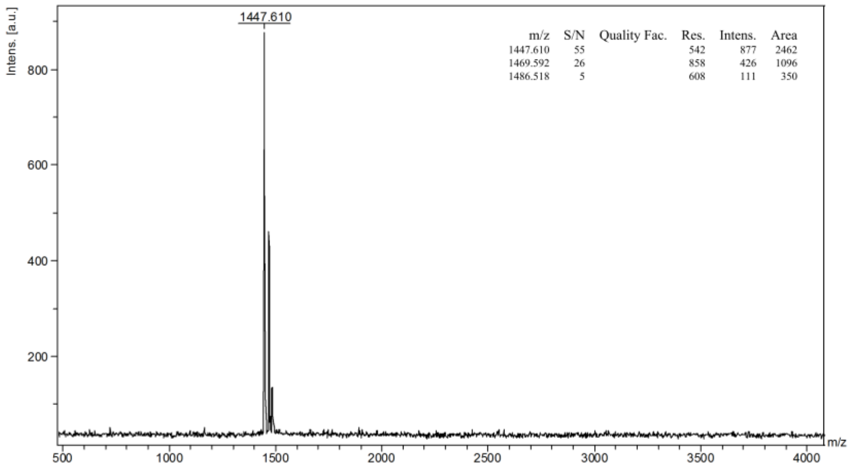

(C_{10})[4]
Mw: 1487.82 g/mol
[MH+]: 1489.071

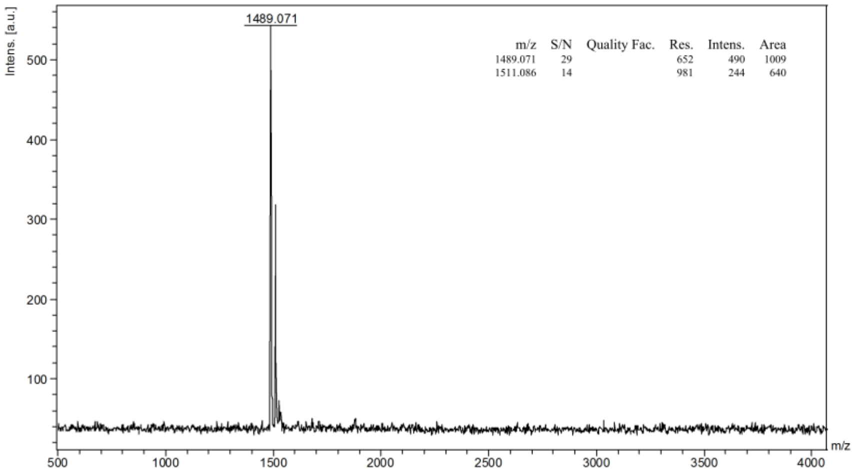

(C_{4})[5]
Mw: 1280.50 g/mol
[MH+]: 1281.631

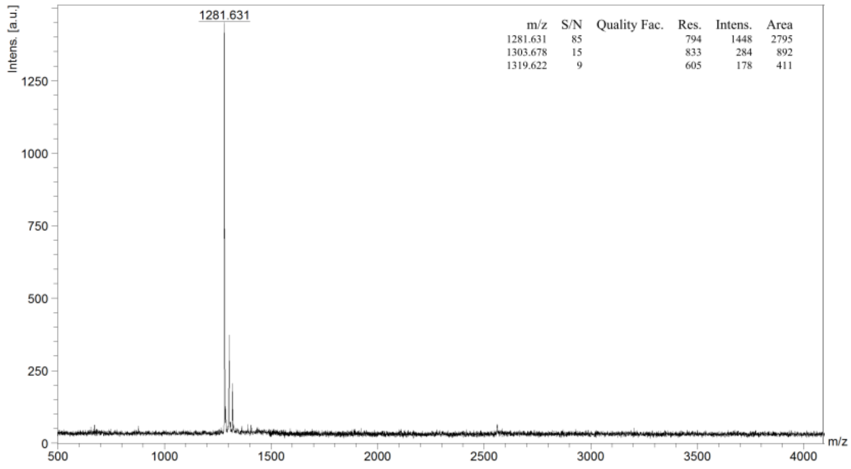

$(C_6)^5$
Mw: 1308.55 g/mol
[MH^+]: 1309.657

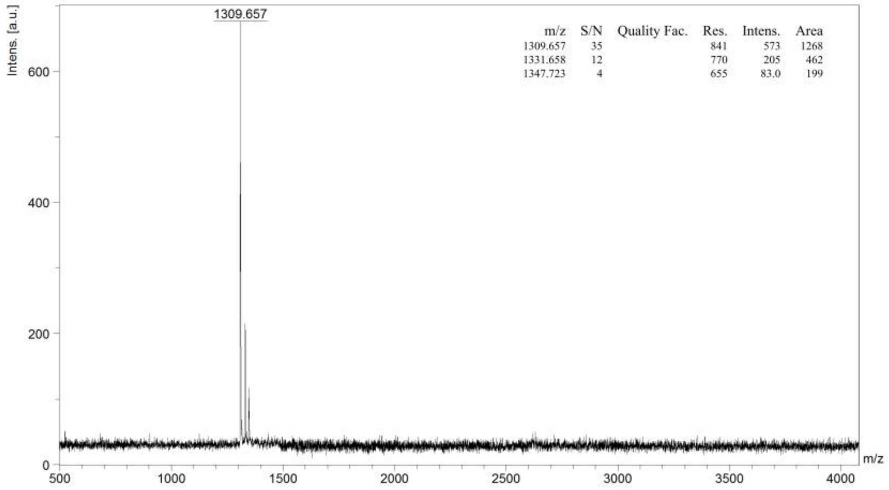

$(C_8)^5$
Mw: 1336.61 g/mol
[MH^+]: 1337.765

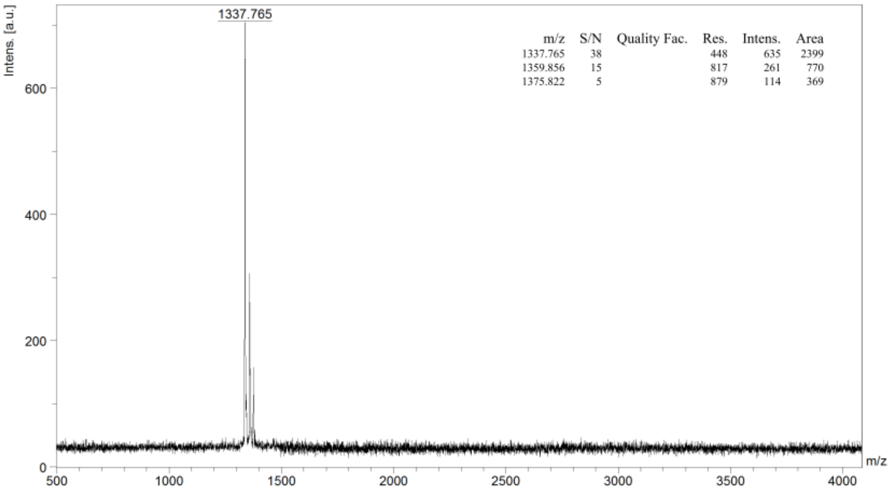

$(C_{10})^5$
Mw: 1364.66 g/mol
[MH^+]: 1365.575

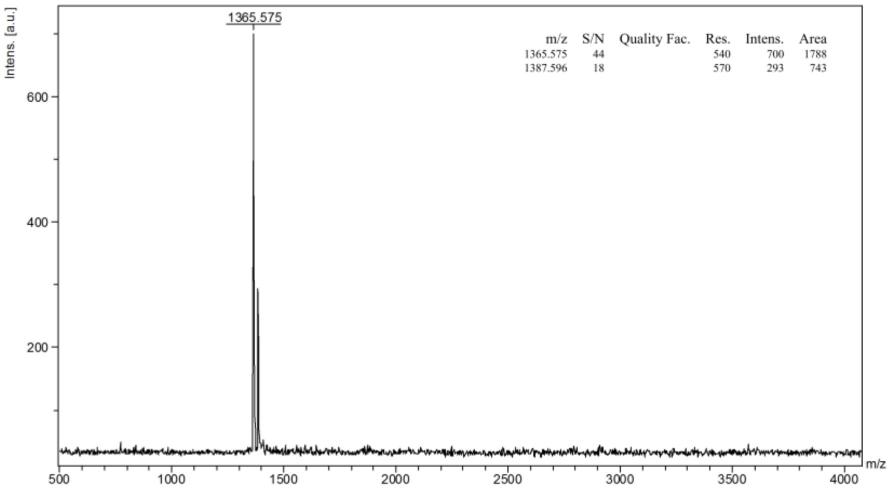

$(C_{12})^{5}$
Mw: 1392.72 g/mol
[MH+]: 1393.785

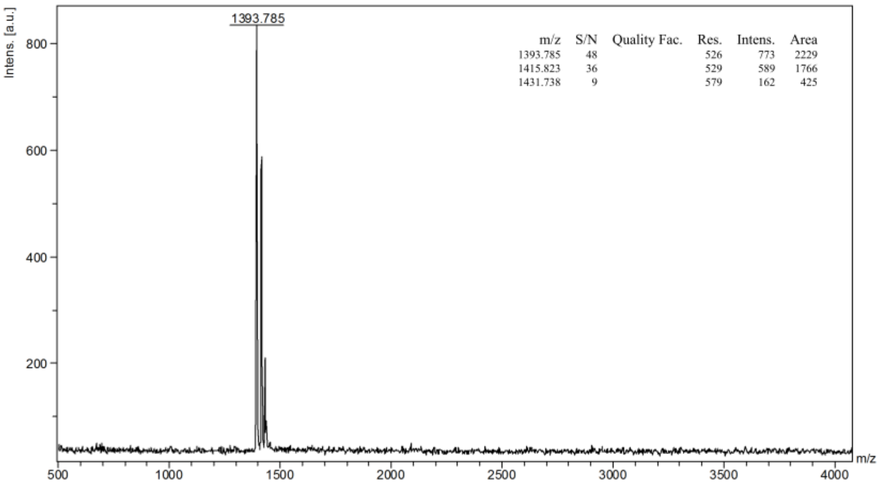

$(C_{14})^{5}$
Mw: 1420.77 g/mol
[MH+]: 1421.534

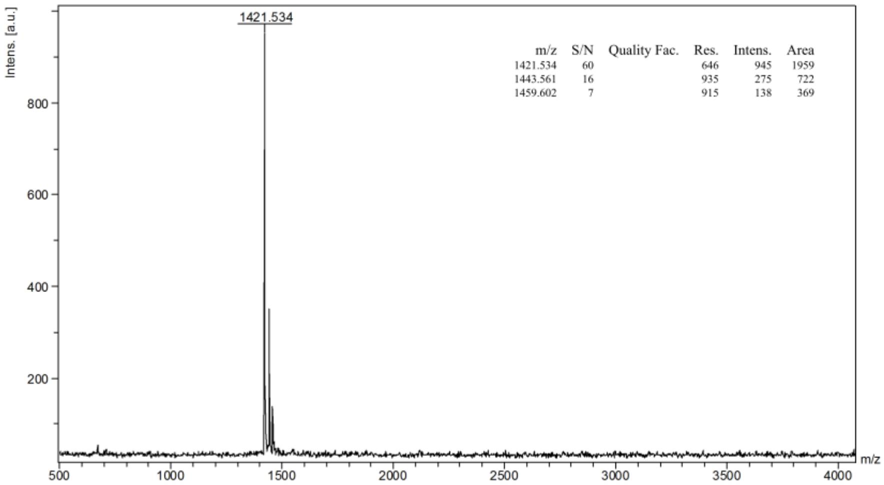

$(C_{4})^{7}$
Mw: 1306.54 g/mol
[MH+]: 1307.750

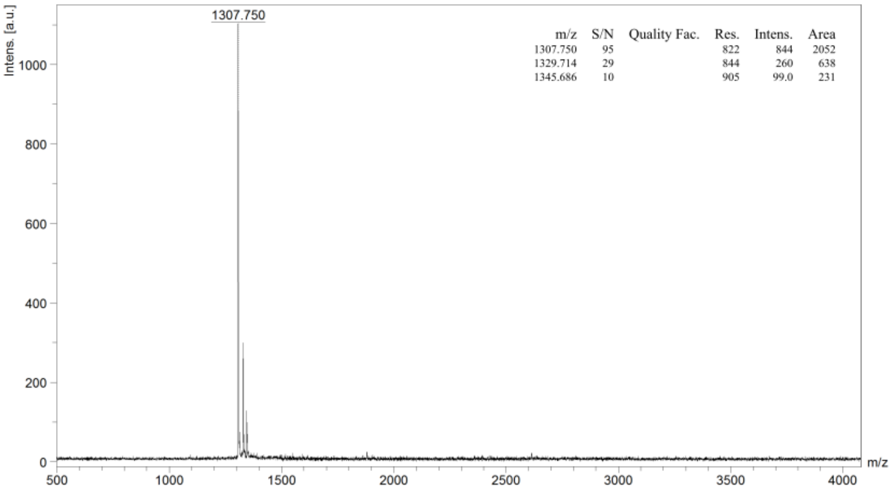

$(C_6)^6$
Mw: 1334.59 g/mol
$[MH^+]$: 1335.280

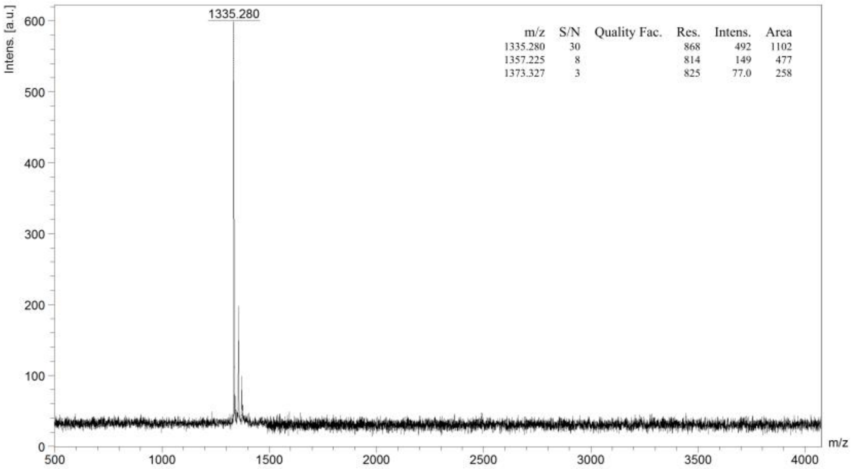

$(C_8)^7$
Mw: 1362.65 g/mol
$[MH^+]$: 1363.683

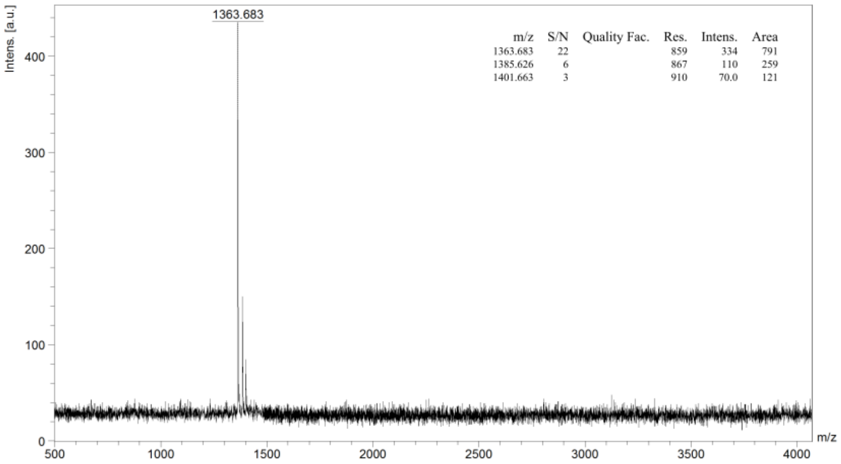

$(C_{10})^7$
Mw: 1390.70 g/mol
$[MH^+]$: 1391.426

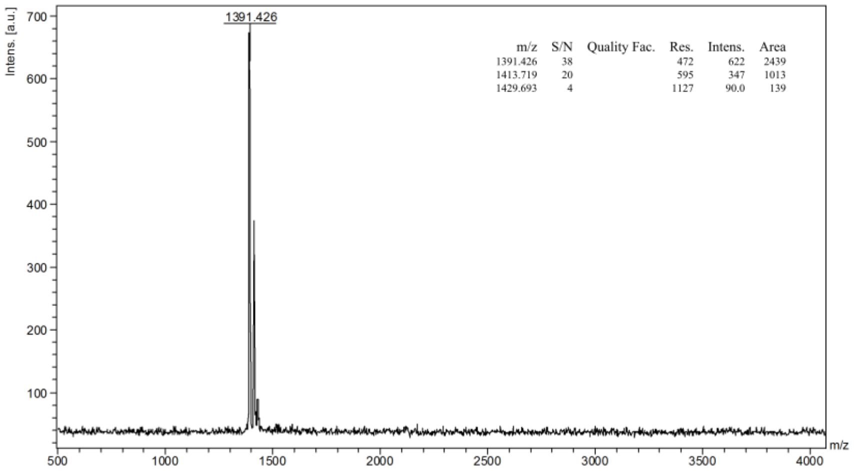

(C12)[7]
Mw: 1418.75 g/mol
[MH+]: 1419.600

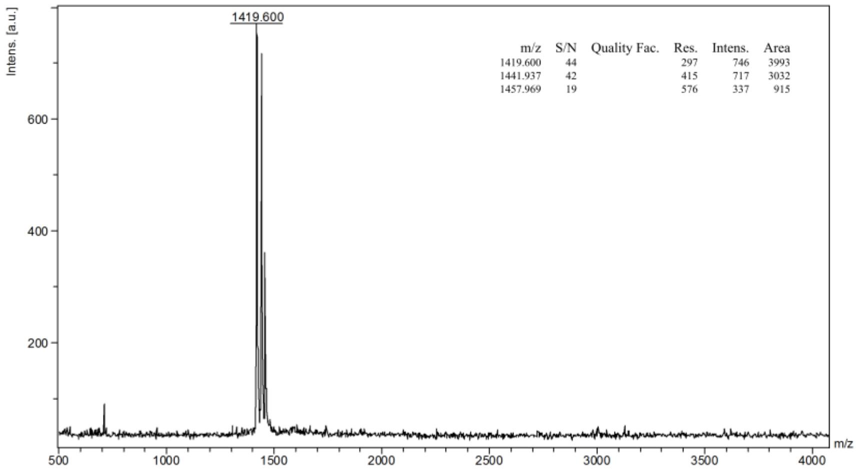

(C14)[7]
Mw: 1446.81 g/mol
[MH+]: 1447.741

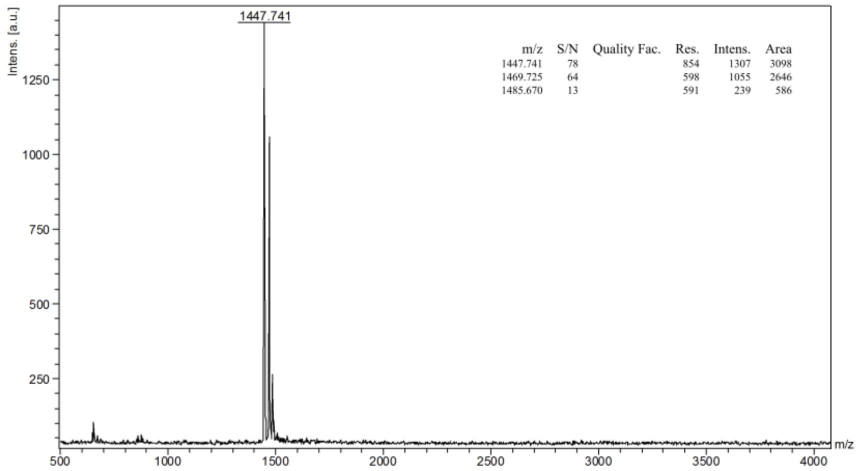

(C10)[8]
Mw: 1487.82
[MH+]: 1488.890

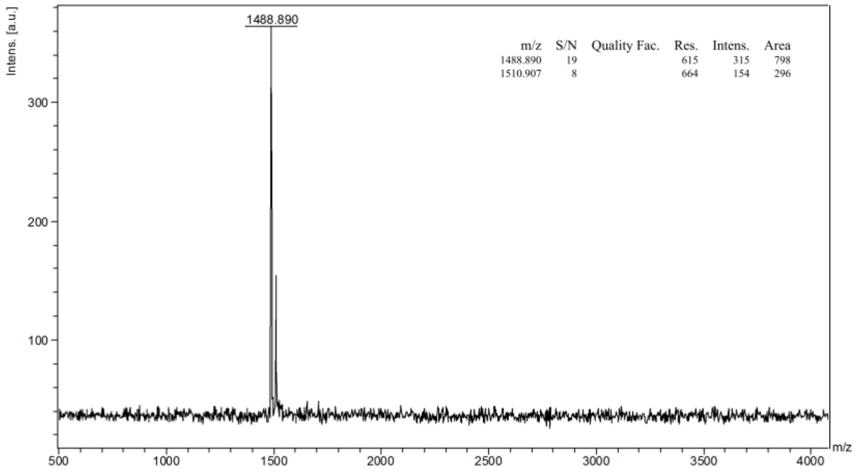

S3(B)
Mw: 1333.68
[MH+]: 1332.910

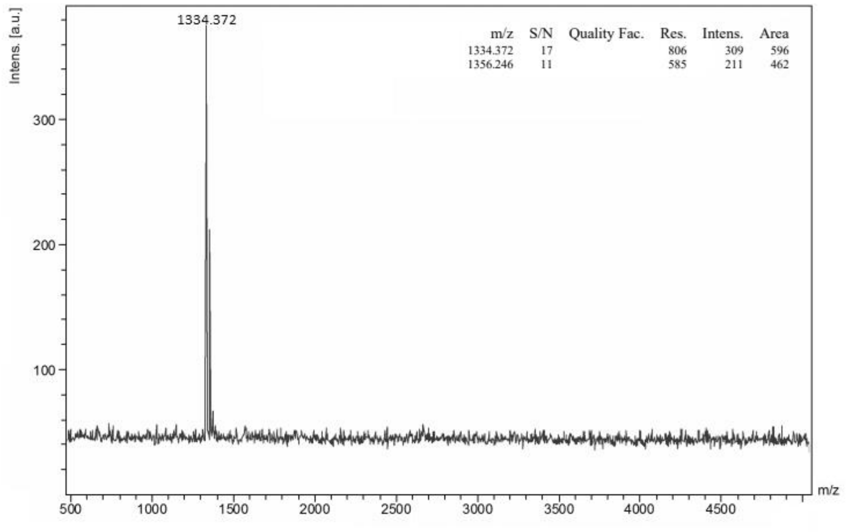

m/*z*: mass/charge ratio; S/N: Signal/Noise ratio; Mw: Molecular weight.
